# Prion protein PrP nucleic acid binding and mobilization implicates retroelements as the replicative component of transmissible spongiform encephalopathy

**DOI:** 10.1007/s00705-020-04529-2

**Published:** 2020-02-05

**Authors:** Richard Lathe, Jean-Luc Darlix

**Affiliations:** 1 Division of Infection Medicine, University of Edinburgh School of Medicine, Edinburgh, UK; 2 Shemyakin and Ovchinnikov Institute of Bioorganic Chemistry, Russian Academy of Sciences, Pushchino, Moscow, Moscow Region Russia; 3 Faculté de Pharmacie, Centre Nationale de la Recherche Scientifique (CNRS) Laboratory of Bioimaging and Pathologies (Unité Mixte de Recherche 7021), Université de Strasbourg, Illkirch, France

## Abstract

The existence of more than 30 strains of transmissible spongiform encephalopathy (TSE) and the paucity of infectivity of purified PrP^Sc^, as well as considerations of PrP structure, are inconsistent with the protein-only (prion) theory of TSE. Nucleic acid is a strong contender as a second component. We juxtapose two key findings: (i) PrP is a nucleic-acid-binding antimicrobial protein that is similar to retroviral Gag proteins in its ability to trigger reverse transcription. (ii) Retroelement mobilization is widely seen in TSE disease. Given further evidence that PrP also mediates nucleic acid transport into and out of the cell, a strong case is to be made that a second element – retroelement nucleic acid – bound to PrP constitutes the second component necessary to explain the multiple strains of TSE.

## Introduction

A growing body of data suggests that the prion theory is incomplete and that the disease-specific form of the prion protein PrP deposited in TSE brain may not itself be the sole infectious villain. This paper aims to reconcile the existing data. Starting with a brief overview of prion theory and its limitations, the biochemical properties of the PrP protein are revisited, notably the overlap between the nucleic-acid-binding/condensing, membrane-binding/inserting, and antiviral activities of PrP, which suggests that PrP and its processing products are antimicrobial proteins (AMPs). The robust reverse transcription (RT) chaperoning activity of PrP and evidence that TSEs are accompanied by the mobilization of diverse retroviruses and retroelements suggest that TSE may involve retroelements. Retroelement nucleic acids associated with PrP could underlie the different strains of TSEs that the protein-only theory fails to explain fully. Although controversial, the notion that PrP associates with nucleic acids is of importance to explain the unusual properties of the infectious agent. The interested reader is referred to earlier reviews and opinions on the same topic (references [1–5] and further references in the text).

## Prion disease – compelling evidence against the protein-only theory

TSEs are a group of neurodegenerative diseases that includes scrapie in sheep, bovine spongiform encephalopathy (BSE) in cattle, transmissible mink encephalopathy, chronic wasting disease of elk and deer, and Creutzfeld–Jakob disease (CJD) in humans. The socioeconomic impact of TSEs is illustrated by the BSE epidemic in 1990–1995, during which 4.4 million cattle were culled in the UK alone [6].

The transmissibility of scrapie by experimental inoculation was first demonstrated by Cuillé and Chelle [7], soon followed by transmission to goats and other species (reviewed in reference [8]). Transmission of CJD to chimpanzees was later demonstrated by Gajdusek and colleagues [9] (see also reference [10]). The archetypal features of TSEs, brain vacuolization and the presence of aggregated protein deposits, have been recognized for over a century (discussed in references [11–13]), although in some cases clinical disease can emerge in the absence of detectable proteinaceous aggregates (see below). The detection of disease-specific amyloid-like plaques [14] and fibrils [15] was followed by the demonstration that these aggregates copurify with infectivity and, importantly, that a major component of these aggregates is a protease-resistant 27–30 kDa form of the host protein PrP [16–20], dubbed PrP^Sc^ after the archetypal disease, scrapie, a refolded product of the native cellular precursor protein, PrP^C^, that is encoded by the *PRNP* gene in humans and by *Prnp* in mice.

PrP has been ascribed multiple functions, ranging from synaptic plasticity to cell-surface signaling, cell–cell communication, and RNA metabolism (reviewed in references [21, 22]). However, laboratory-raised *Prnp*-mutant mice display only subtle deficits, often irreproducible, in part because in four of six lines the knockout led to pathogenic upregulation of the adjacent Doppel (*Prnd*) gene, explaining many discrepancies (reviewed in reference [23]). Indeed, there has been little consensus about the physiological role of PrP, and its primary function has remained elusive.

The ‘prion’ or ‘protein-only’ theory, as advocated by Prusiner and others (e.g., [24]), holds that the cellular form of the protein, PrP^C^, undergoes a conformation change, generating the ‘scrapie-specific’ form PrP^Sc^. (In the text we use the terms ‘protein-only’ and ‘prion’ theory interchangeably to refer to the concept that the infectious agent lacks an informational molecule such as a nucleic acid, but we do not exclude protein post-translational modifications and/or the presence of bound non-informational molecules such as lipids.) In turn, PrP^Sc^ binds to PrP^C^ and promotes Prp^C^ → PrP^Sc^ conversion, leading to amplification of (supposedly neurotoxic) PrP^Sc^ and disease (see references [25–27] for review). In support, other than PrP itself, no other agent has been routinely detected in infectious fractions purified from diseased brain. The agent appears to be resistant to treatments that normally inactivate nucleic acids, and if a nucleic acid is associated with PrP, it has been argued to be short [28], excluding a conventional viral genome.

However, several lines of evidence suggest that the prion theory is incomplete, and other data argue that a nucleic acid component may be obligatory for infection: first, the existence of multiple strains of the agent and the phenomenon of strain competition; second, the paucity of infectivity of the recombinant prion protein, and third, evidence pointing directly to a nucleic acid component associated with the protein.

## TSE strains – too many to underlie a protein-only hypothesis

The small size of the agent suggests that it might be able to replicate without nucleic acid [29–31], leading to the ‘prion’ hypothesis [24, 32] of an infectious polypeptide. However, ever since the very first studies on scrapie it was evident that there are multiple strains of TSE that differ in host-specificity, replication rate and incubation period, type of brain pathology, end-point titer, strain stability, and resistance to inactivation [33, 34], irrespective of host *Prnp* genotype. Alan Dickinson and colleagues [2, 33] described multiple different strains, and Moira Bruce [34] referred to 20 strains and summarized the different properties of 14 mouse-adapted strains. To these one can add at least three more recent BSE-derived strains [35], two strains of hamster scrapie (hyper and drowsy) isolated following inoculation with transmissible mink encephalopathy [36], at least two strains of chronic wasting disease in deer, elk, and moose [37], and multiple types of human TSE, including at least two types of CJD [38], as well as fatal familial insomnia (FFI), Gertsmann–Sträussler–Scheinker syndrome (GSS), and Kuru, which may themselves have subtypes, making a total of at least 32 strains. In this respect, the agent resembles a virus (e.g., there are more than 30 subtypes of human papillomavirus).

Moreover, TSE strains can undergo mutational change that alters their properties [34]. None of these observations are easily explained by the protein-only hypothesis. Bruce and Dickinson stated: ‘The considerable strain diversity in scrapie, together with the evidence for mutational change {…}, offer compelling arguments that scrapie has its own independent replicating genome’ [2].

## Strain competition

Strain competition affords a further complexity. Some TSE agent strains are ‘fast’ (such as scrapie isolate 22A), producing early pathology, whereas others are ‘slow’ (such as isolate 22C); Dickinson and colleagues explored whether inoculation with the slow agent might interfere with later superinfection by the fast agent. Perhaps surprisingly, preinoculation of mice with the slow agent, followed 30 days later by the fast agent, led to a highly significant delay in fast-agent pathology [39]. Indeed, a slow agent can block pathogenesis so effectively that the later-inoculated fast agent appears to take little active part in the disease [40]. Strain competition has been confirmed both *in vivo* and *in vitro* [41, 42].

The mechanism is so far unknown. Dickinson and colleagues suggested that there might be only a limited number of ‘replication sites’, which the slow isolate blocks, and that the production of new sites must be infrequent [39, 40]. Manuelidis raised the intriguing possibility that the slow agent might produce defective interfering particles (DIPs) [41]. Traditional DIPs emerge as genome-deleted variants of diverse virus types and compete with the parent virus for replication, but without themselves causing pathology, thus markedly slowing the disease process (see references [43, 44] for recent literature). A canonical example is afforded by lymphocytic choriomeningitis virus (LCMV). Infection of neonatal rats causes severe cerebellar pathology, but coinfection with LCMV DIPs is able to slow or abolish discernable disease development [45].

According to the DIP model [41], defective particles produced by the slow TSE strain would swamp replication sites, blocking propagation of the fast strain. This is an attractive model. For TSEs, the site of competition (‘replication site’) is not known, but Dickinson [46] has argued that PrP^C^ is itself the limiting target, and there is evidence that PrP^C^ abundance declines during the course of infection [47, 48].

Strain competition is therefore not necessarily inconsistent with the prion (protein-only) theory but does require a defined PrP:PrP interface that a slow strain can occlude.

## PrP structure is incompatible with multiple stable configurations

The protein-only theory seeks to explain TSE strains by multiple alternative configurations of aggregated forms of PrP. PrP can undergo a transition from a globular form to an aggregated β-rich structure (see below), but this switch is not consistent with multiple stable alternative 3D structures.

Yeast prion proteins (e.g., Ure2p and Sup35p) are widely cited as a precedent for generating multiple configurations that can be propagated across cell division. In yeast, the multiple protein forms are generated by a characteristic glutamine/asparagine-rich (poly/Q/N) primary sequence that leads directly to alternatively stacked amyloid-like β-sheet structures. Some variants can persist over multiple passages, although the variant-specific properties of others can be lost within a single passage (see reference [49] for review). In these proteins, this polyQ/N region (‘prion domain’) is essential for the protein to switch to alternative stable and heritable prion conformations (e.g., [50]). By contrast, pWALTZ/PrionW analysis (http://bioinf.uab.cat/prionw/ [50, 51]) reveals that mammalian (mouse, sheep, bovine, human) PrP proteins entirely lack any such yeast-type prion domain. Thus, although this conclusion relies on the design of these bioinformatic tools, we suggest that yeast prions may not afford a precedent for TSE strains.

It is not impossible that an alternative type of protein configuration remains to be discovered that is capable of generating multiple stable configurations, but so far there is no adequate explanation for the multiple strains of TSE. Different TSE strains do display discrete conformational differences in PrP-derived molecules (perhaps consistent with a tightly bound second component, see below), but advocates of the protein-only theory point instead to subtle changes in the conformation and post-translational modification of PrP protein (e.g., [52–54]), although without clarifying what interactions might cause the differential structural modifications of the identical substrate protein – and how these could stably propagate to generate the 30 or more distinct strains of TSE.

A further critique of the conformation hypothesis is that infectivity resides in complexes of at least 10–20 PrP^Sc^ molecules, and not in PrP^Sc^ monomers to pentamers [55]. This is consistent with the PrP:nucleic acid sequestration hypothesis (see below) but does not favor the prion hypothesis because it would require a protein conformation that is present in (PrP^Sc^)_10–20_ but absent from (PrP^Sc^)_2–5_.

In addition, the prion theory relies on a defined conformation of PrP^Sc^, and subtle structural differences therein, to explain the different strains of agent that are inferred to propagate via a protein → protein conversion mechanism. However, this is potentially problematic because the key N-terminal region of PrP^C^ is intrinsically disordered (discussed later), which would tend to preclude the generation of stable (and transmissible) conformational variants. Indeed, disease, infectivity, and strain identity can be associated with soluble forms of PrP (in the absence of PrP^Sc^) that have a poorly defined structure.

## Dissociation between PrP and infectivity: PrP^Sc^ alone is poorly infectious

Multiple studies have reported that high levels of infectivity can be present in the absence of detectable PrP^Sc^ [56–60] and, conversely, that high levels of PrP^Sc^ can be present in the absence of infectivity (e.g., [61]). Importantly, TSE infectivity appears to expand rapidly following infection, but the generation of PrP^Sc^ only follows after a delay (reviewed in reference [5]). Centrally, highly purified PrP^Sc^ is poorly if at all infectious.

PrP^Sc^ that has been biochemically purified from infected brain requires at least 2 × 10^3^ to 10^6^ molecules of PrP^Sc^ for infectivity, and sometimes more [55, 62–64]. These reports are not easily consistent with the concept that PrP^Sc^ is itself the infectious agent.

Recombinant PrP molecules, even if aggregated into protease-resistant analogs of PrP^Sc^, are generally non-infectious (e.g., [65, 66]). Collinge and colleagues evaluated 20,000 different *in vitro* conditions, and in no case were they able to generate infectivity from recombinant PrP [67]. By contrast, Legname *et al.* [68] reported induction of disease by inoculating amyloid-like aggregates of recombinant PrP. However, in this case, the recipient transgenic mice were incipiently disease-prone because they expressed very high levels of mutant PrP, and, before passage, the same inocula failed to produce disease in wild-type mice.

Serial protein misfolding cyclic amplification (sPMCA) has been employed to generate large amounts of PrP^Sc^ from a recombinant seed *in vitro*, and in some cases the material generated was reported to cause disease following intracerebral inoculation [69, 70]. However, the observed titers were extremely low, again arguing that PrP^Sc^ alone is unlikely to be the infectious agent. In other systems, no infectivity was reported with recombinant PrP despite the presence of large amounts of protease-resistant PrP^Sc^ [71].

To achieve significant infectivity has required cyclic refolding in the presence of whole-brain extract [72–74] or an excess of total liver RNA [69, 75], leaving open the possibility of a second component. Even so, titers have been low and, moreover, in some cases, strain differences disappeared on amplification [76, 77].

The most recent reports systematically rely on brain homogenate or on brain or liver RNA to generate infectivity [75, 78, 79]. For the most part, only low levels of infectivity were generated. For example, Wang *et al.* [79] reported that 10^8^ molecules of PrP^Sc^ generated *in vitro* were required for infectivity. Burke *et al.* [80] showed that PrP^Sc^ can be generated *in vitro* by cyclic refolding amplification in the absence of cofactors, but the PrP^Sc^ generated was not infectious – they state, ‘To our surprise, the bioassay results were completely negative’. Infectivity could only be generated by reamplification in the presence of brain homogenate/extracts, demonstrating once again that a second component is essential.

One report stands out: Deleault *et al.* reported the generation of infectivity from recombinant PrP amplified in the presence of high (millimolar) concentrations of phosphatidylethanolamine (PE), whereas the equivalent reaction product produced in the absence of PE was not infectious [77]. Why some preparations are infectious, whereas those produced by a slightly different protocol are not, remains mysterious (discussed in reference [81]), pointing to a so far unknown alternative infectious conformation, another component, or both. This new conformation would need to operate above and beyond the supposed alternative conformations mooted to explain the 30 or more strains of the agent. However, the biochemical purity of the PE employed may be debatable ([82] as the cited source), and other researchers have reported that PE inhibits prion replication [83]; the generation of *de novo* infectivity in the presence of PE alone requires independent validation.

To our mind, the best interpretation so far is that of Timmes *et al.*, who, to explain the circa 10^5^-fold difference in infectivity between *in vitro* PrP^Sc^ and *in vivo* PrP^Sc^, proposed that a ‘stochastic event’ [71], possibly taking place *in vivo* following inoculation, is essential before *de novo* infectivity can be generated from recombinant molecules. Schmidt *et al.* [67] reached the same conclusion.

In other words, this leaves open the possibility that large quantities of modified PrP, inoculated directly into the brain, might sporadically and at low frequency recruit an endogenous agent for disease propagation. This contrasts with ‘wild’ transmission in sheep, which is thought to involve contact between lambs and placenta from infected ewes, and notably, blood contact via scratching posts [84] – the behavior that names the disease [85]. Until transmission of disease by purified recombinant PrP^Sc^ in the absence of cofactors has been demonstrated to take place by equivalent routes in animal models (oral, cutaneous), one must remain open to the possibility that a protein conformation, alone, might not be the transmissible agent in natural scrapie.

## Evidence for a crucial nucleic acid component in TSE

Inactivation studies argue that the agent cannot comprise a nucleic acid component (or at least one of genome size); however, alkali treatment (pH 10 for 1 h at 4 °C) reduced agent titer by a factor of 1000) [24], consistent with an RNA component. Because ribose (unlike deoxyribose) has adjacent 2’,3’ hydroxyls, exposure to high pH leads to chain scission, whereas DNA (and protein) is largely refractory to alkali. In fact, the TSE agent appears to be significantly more sensitive to alkali that a control RNA viroid-based pathogen (potato spindle tuber viroid [86]), suggesting the presence of an obligate RNA component. In addition, Riesner and colleagues reported that UV irradiation at 254 nm (which principally targets nucleic acids) reduced infectivity by a factor of 1000 [87].

Although purified RNA from diseased brain is not itself infectious (e.g., [66, 88]), it is not impossible that this failure is because brain abundantly expresses the atypical RNase 1 (also known as brain ribonuclease, BRB) that, unusually, can degrade dsRNAs (2000-fold more efficiently than RNase A) and is also induced by dsRNA [89]. A protective component (protein, lipid, other) may be vital to prevent rapid degradation of exogenous RNA.

PrP is a strong contender as a protective shield for RNA. Early studies argued that the agent is resistant to nucleases, but PrP is an RNA-binding protein, and bound RNA can precipitate PrP^C^ to PrP^Sc^ conversion (see below); indeed, PrP-mediated aggregation can protect bound nucleic acids against degradation (as observed *in vitro*; J-L.D., unpublished). Solubilization and nuclease digestion of released nucleic acid has been shown to abolish the infectivity of purified brain fractions while leaving PrP^Sc^ intact, again arguing for a nucleic acid component [90] – probably RNA, given the alkali sensitivity of the agent. Conversely, in some reports, aggressive removal of PrP proteins by proteinase digestion failed to reduce infectivity (e.g., [91, 92]).

Studies over several years, notably by Detlev Riesner and colleagues, have carefully examined infectious fractions for nucleic acids. In the most recent report, using return refocusing electrophoresis, nucleic acids in the range of 25 nt were detected, although there was evidence for larger molecules in the 100–400 nt range [87] (note that specific fragments as short as 25 nt can be unique in the mammalian transcriptome [93]). However, to put this into perspective, if circa 10^6^ molecules of PrP^Sc^ are required for infection (see earlier), detecting the ‘one in a million’ specific bound nucleic acid that might be responsible for infectivity represents a major challenge.

By contrast, in direct support of a nucleic acid component, Simoneau and colleagues demonstrated that neither purified PrP^Sc^ nor small RNA fragments derived from scrapie-infected brain were able to establish infection, but 5 of 24 animals inoculated with the combination of the two fractions succumbed to a scrapie-related disease [66, 94].

In sum, the existence of 30 or more stably propagating strains of disease, combined with the alkali and UV sensitivity of the agent, the key role of RNA in cyclic reamplification of infectivity, and the requirement for RNA to generate infectivity from isolated components, suggests that something else in infectious fractions, perhaps nucleic acid, might confer strain properties. We address below the nucleic-acid-binding properties of PrP with a view to casting light on the possible identity of the missing component.

## PrP is a nucleic acid-binding protein

### Conserved polybasic regions in the N-terminal domain of PrP

**Fig. 1:**
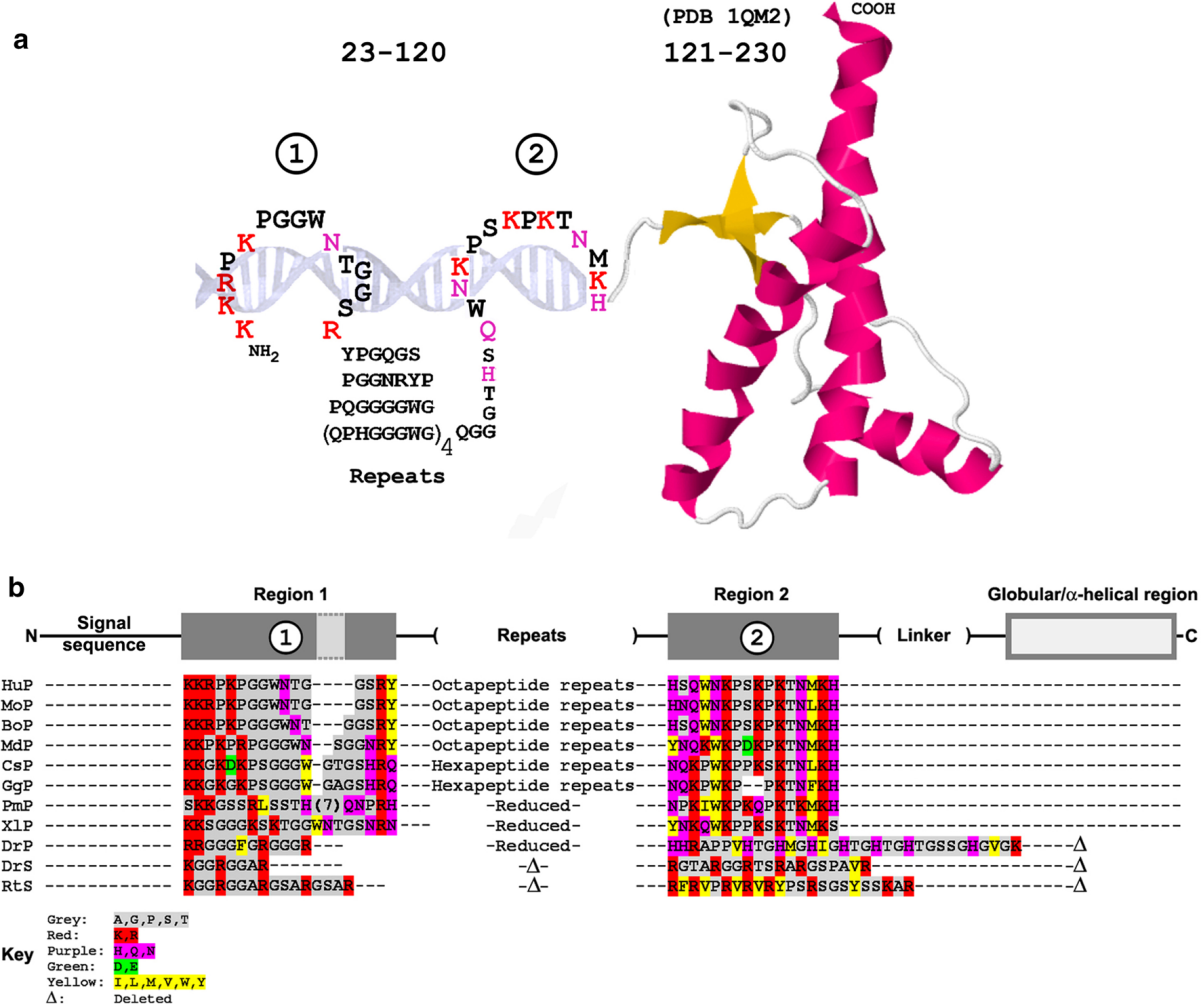

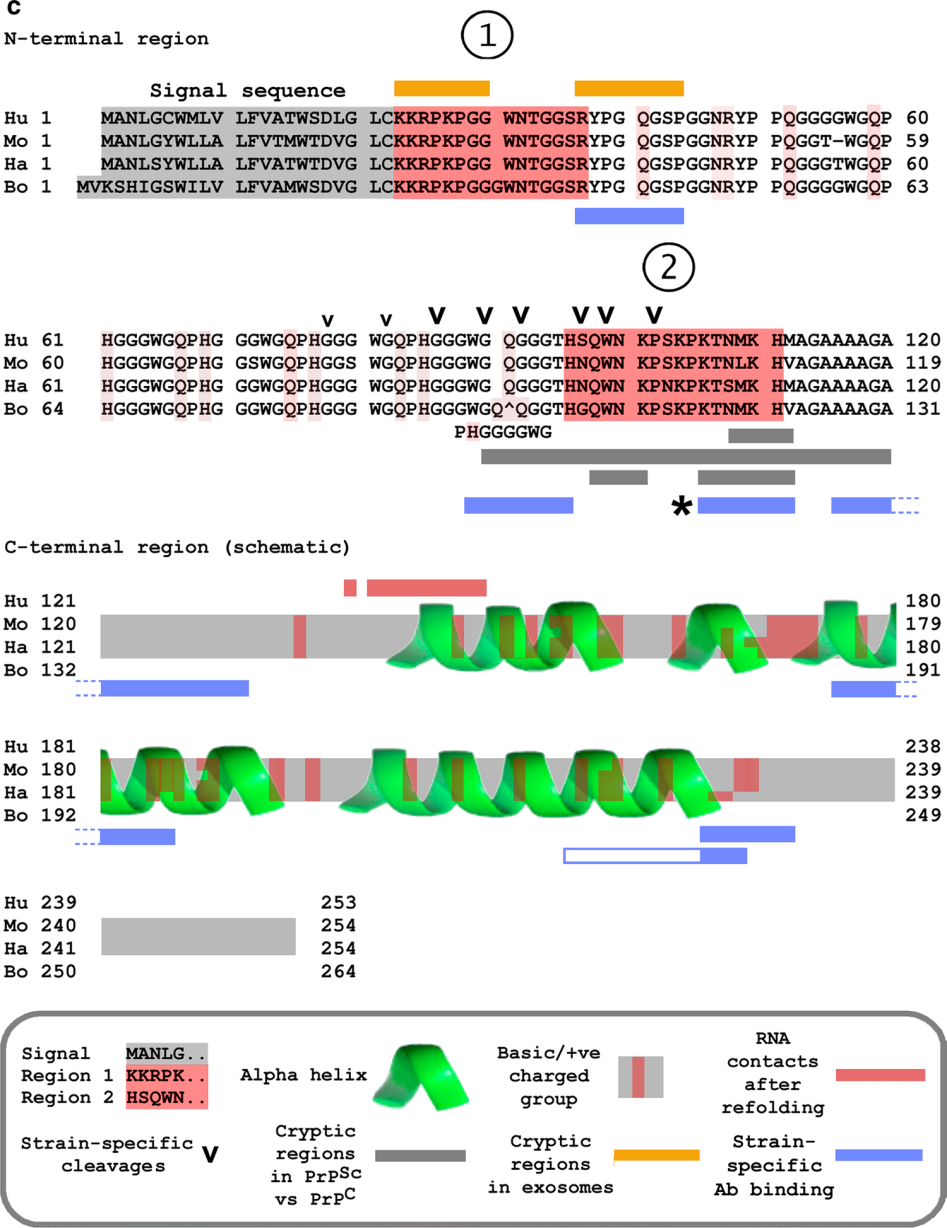
Two basic regions in the N-terminal segment of PrP and its immediate evolutionary precursors: TSE strain-specific structural differences. (A) The two polybasic regions in the intrinsically disordered N-terminus of human PrP, schematically depicted in complex with a nucleic acid (DNA for illustration), are shown fused to the globular/α-helical region of the protein established by NMR (PDB 1QM2 [236]). (B) Conservation of two basic regions in PrP and its evolutionary precursor (Shadoo/SPRN). PrP sequences (P) are Hu, human; Mo, mouse; Bo, bovine; Md, opossum (marsupial, *Monodelphis domestica*); Cs, mousebird (*Colius striatus*); Gg, chicken (*Gallus gallus*); Pm, viper (*Protobothrops mucrosquamatus*); Xl, African clawed frog (*Xenopus laevis*). The final three entries are prion protein 1 from zebrafish (DrP, *Danio rerio*), Shadoo/SPRN from zebrafish (DrS), and Shadoo/SPRN from whale shark (RtS, *Rhincodon typus*) which lack the C-terminal globular region and have alternative region 2 polybasic sequences (His-rich in DrP, and Arg-rich in DrS and RtS). Color code: red, highly basic (Lys/Arg); violet, basic (Asn/Gln/His); green, acidic (Asp/Glu); yellow, hydrophobic (Leu/Ile/Val/Met/Phe/Tyr). (C) TSE strains and the N-terminal region of PrP. Summary of strain-dependent cleavage sites, strain-specific antibody binding, occlusion of antibody binding, and RNA-dependent refolding of PrP. The figure shows the alignment of the N-termini of human (Hu), mouse (Mo), hamster (Ha), and bovine (Bo) PrP^C^ sequences (the C-terminus is depicted schematically). Regions 1 and 2 are as in panels A and B. The exact details depend on the host species (and host genotype) as well as on the strain of the agent. Strain-specific cleavages (v) are sites where high-resolution mapping (e.g., mass spectrometry) demonstrates that the precise sites of PrP proteolytic processing differ significantly between infections with different strain types [119, 128, 129, 237]. Grey and brown horizontal bars show regions that are occluded (‘cryptic’) in PrP^Sc^ versus PrP^C^ (grey) and that can also differ according to the strain of TSE [130–133], or that are occluded in exosomes from scrapie-infected cells (brown) [220]. Blue horizontal bars indicate epitopes for strain-specific antibody binding [238–240]. The epitope marked with an asterisk (*) is recognized by antibody 3F4 [241]; RNA binding to PrP *in vitro* occludes 3F4 binding (J-L.D, unpublished). Molecular dynamics simulations indicate that RNA bound to site 1 can lead to refolding of the polypeptide and dissolution of the first α-helical region; predicted new contacts after refolding are indicated by red bars; there may be further contacts within PrP [115]. ‘Basic’ amino acids indicated in the figure include not only K and R but also H, Q, and N, which contain positively charged groups with potential to interact with nucleic acid phosphates. The panel aims to highlight strain-specific differences and is not intended as a comprehensive survey

Mammalian PrP protein comprises two structurally distinct components. The N-terminus is an intrinsically disordered region (IDR), a characteristic of many RNA-binding proteins that regulate RNA functions from transcription to maintenance [95, 96], whereas the C-terminal region of native PrP adopts a largely globular/α-helical conformation (Fig. 1A). Disease is accompanied by aggregation of PrP and transition of the globular C-terminus from an α-helical conformation to a β-sheet [97, 98].

At least three *PRNP*-like genes are present in the human genome: *PRNP*, *SPRN* (Shadoo/shadow of prion protein), and *PRND* (Doppel). The *SPRN*-like genes are thought to be the immediate evolutionary precursors to extant *PRNP*, and although these retain the N-terminal region, these lack the C-terminal globular domain of the protein (Fig. 1B) [99–101]. Sequence analysis (Fig. 1) identifies two polybasic regions in the N-terminal IDR that have charge complementarity to polyacidic nucleic acids, and these regions are substantially conserved between humans and amphibians (*Xenopus laevis*). The evolutionary antecedent to PrP protein, Shadoo, is also an RNA-binding protein by virtue of the conserved basic motifs (RGG box, region 1 in Figure 1) in the N-terminus of the protein [102], which are also present in PrP as well as in other RNA-binding proteins such as FMRP [102]. Direct binding of nucleic acid to Shadoo has been confirmed [103]. Interestingly, the N-terminal regions of Shadoo-like proteins of earlier representatives of the vertebrate lineage such as whale sharks (*Rhincodon typus*) and zebrafish (*Danio rerio*) also contain a second basic region, as in PrP, but of different composition. In zebrafish ‘prion protein 1’ the second basic region (region 2) is replaced by a histidine-rich motif, whereas in sharks, this is a distinct but also highly basic arginine-rich motif (Fig. 1B).

The evolutionary conservation of two polybasic regions in the N-terminus of PrP family proteins demonstrates that the inherent affinity of PrP for nucleic acid has been retained since the beginning of the vertebrate lineage, arguing that this feature is likely to be central to the present-day function of mammalian PrP protein.

### Nucleic acid binding by PrP

There is direct evidence that PrP binds to nucleic acids ([104, 105], reviewed in references [3, 106, 107]) via its N-terminus. Nucleic acid binding takes place *in vivo*: PrP protein could be affinity purified from TSE brain (CJD, BSE, scrapie) using either anti-DNA antibody or single-stranded DNA-binding protein from an *E. coli* bacteriophage [108]. Of note, the nucleic-acid-binding repertoire of PrP *in vivo* may be extended because (i) PrP is prone to dimerize [109], potentially providing multiple binding sites in the dimer, and (ii) PrP may also interact with other nucleic-acid-binding proteins – the most significant hits in a microarray screen for PrP binding partners were RNA-binding proteins [110].

### Key role of the disordered N-terminal region of PrP: nucleic acid binding promotes sequential refolding

The intrinsically disordered N-terminal region of PrP is necessary for infectivity. Deletion of basic region 1 leads to an apparent large reduction in infectious titer as well as a ~ 75% reduction in agent replication rate [111, 112] (as assessed by the increase in incubation period), and deletions extending into region 2 abolish infectivity propagation (reviewed in reference [113]).

Nucleic acid binding to the N-terminal region can induce refolding of the PrP molecule. To illustrate, the addition of nanomolar concentrations of DNA to recombinant mouse PrP leads to an increase in turbidity as assessed by light-scattering at 400 nm. The change is rapid, with a latency period of ~ 3 minutes as revealed by the fluorescence kinetics of a bound reporter [114]. Molecular dynamics simulations indicate that RNA docked to the region 1 polybasic region leads to dissolution of the first α-helix in the C-terminal region [115]; this is thought to lead towards the formation of large fibrillar ribonucleoprotein complexes, for example as demonstrated by electron microscopy (e.g., [116]), in which the C-terminal region adopts an extensive β-sheet structure [97, 98].

Although undoubtedly an oversimplification (protein refolding generally requires a series of metastable states), PrP refolding appears to involve (at least) two different configurations that differ in their sensitivity to proteinase K (PK) [117]. In the first step, refolding of PrP^C^ generates a flexible structure that remains susceptible to digestion with PK [118], termed PK-sensitive (s)PrP^Sc^ [119]. In the second step, PrP forms dense aggregates, possibly covalently crosslinked [120], in which the core of the protein is refractory to PK digestion, termed PK-resistant (r)PrP^Sc^. Both forms are associated with infectivity [121]. sPrP^Sc^ resembles in some ways an intermediate form, dubbed PrP*, that was proposed earlier on theoretical grounds to be a precursor to protease-resistant PrP^Sc^ [122], although whether the two forms are equivalent has not been established.

The soluble form, sPrP^Sc^, can undergo an assembly process that generates liquid droplets (also known as proteinaceous membrane-less organelles/coacervates/hydrogels) upon its interaction with RNA. RNA binding by IDRs is crucially important for liquid–liquid phase separation [123], attributed to high local concentrations of negative charges [124]. The IDR not only extends the ligand-capture radius of the protein but also permits refolding of the IDR into an ordered 3D structure in response to ligand binding [125, 126]. Droplets are composed of diverse RNA-binding proteins in association with different RNA species, notably non-coding RNA, undergo liquid–liquid phase transitions, and dynamically assemble and disassemble, and components can exchange with the surrounding liquid phase within seconds to minutes [124].

Droplet formation by PrP^C^ has been confirmed [127]. Moreover, Alzheimer Aβ (discussed later) has been proposed as a key component of PrP-based droplets [127] and in some ways resembles the PrP binding partner, ‘protein X’, that was postulated earlier to play a role in the transition from PrP^C^ to PrP^Sc^ [122]. In sum, there is reason to suspect that the intermediate proteinase-sensitive form of PrP, sPrP^Sc^, represents a dynamic assembly of PrP into liquid droplets following binding to nucleic acid, in association with other RNA-binding proteins, which is then followed by irreversible aggregation to generate rPrP^Sc^.

Of note, the sPrP^Sc^/rPrP^Sc^ ratio in TSE depends on the strain of the agent. Indeed, different strains adopt conformations that differ in protease sensitivity [117, 118]. This meshes with several studies in which proteolytic cleavages in the immediate vicinity of region 2 (arrows in Fig. 1C) differ according to the strain of agent [121, 128, 129], as well as with regions that are occluded (‘cryptic’) in PrP^Sc^ versus PrP^C^ – and that can also differ according to the strain of agent [130–133] (horizontal bars in Fig. 1C). It remains unknown what causes these conformational differences in PrP^Sc^, but it is plausible to suggest that different nucleic acid ligands bound to region 2 might potentially be responsible for differential cleavage of the complex (Fig. 1C). Although direct evidence for this is so far lacking, this possibility has not yet been systematically addressed.

In the following, we focus on an important aspect of the interaction between PrP and nucleic acids: PrP is a defense protein that protects against invasion by extraneous infectious agents.

## PrP is an antimicrobial protein (AMP)

Nucleic acid binding is a central feature of AMPs (see below), a diverse group of evolutionarily ancient proteins that predate the adaptive immune system. These proteins, and often active peptide subfragments generated by proteolytic processing, have potent activity against a wide range of viruses, bacteria, and yeasts, acting via several different pathways, often in parallel (reviewed in references [134–136]).

## Nucleic acid binding is a central feature of AMPs

AMPs are generally held to centrally exert their antimicrobial properties by interacting with membranes. However, membrane phospholipids and sulfated glycosaminoglycans resemble nucleic acids in that they are polyanions, and dual nucleic acid and membrane binding is thus a common feature of AMPs [136, 137]. These dual nucleic-acid- and membrane-binding properties of AMPs are not widely recognized, and we therefore provide two further examples.

The classical AMP LL-37 displays robust nucleic-acid-binding activity [138, 139] and can enter the nucleus and modulate gene transcription. These properties are shared by the AD Aβ peptide, whose antimicrobial activity against a variety of infectious agents, including viruses, bacteria, and yeasts, is well documented (reviewed in reference [140]). Aβ displays the structural signature characteristics of a nucleic-acid-binding protein [141], binds directly to DNA [142–146], and can also enter the nucleus to modulate transcription [147]. Of note, like both LL-37 and Aβ, PrP can also enter the nucleus, where it associates with chromatin [148].

Interestingly, in addition to direct nucleic acid binding (reviewed above), PrP binds tightly to Aβ, PrP and Aβ are codeposited in both AD and TSE brain, and PrP modulates the generation and fibrillization of Aβ (see reference [149] for review), reinforcing the idea that both PrP and Aβ are components of the innate immune system.

## PrP is membrane-active

Like conventional AMPs, PrP can take up a transmembrane configuration [150] and/or insert into membranes [151–153]. Studies on the second basic region of PrP suggest that membrane binding by this region generates membrane pores that penetrate only half of the membrane [154]. To identify the sequences involved, Shin *et al.* used protease digestion of membrane-inserted hamster PrP to identify a protected peptide, NH_2_-NKPSKPKTNMK-COOH, which corresponds to region 2 in Figure 1 [155]. It remains unclear why region 1 was not identified in this assay, but, interestingly, in this paper, a further peptide, also basic in nature, was identified that maps to the globular region of the protein, suggesting that regions downstream of the N-terminus may further contribute to interactions with membranes (and perhaps with nucleic acids) by present-day mammalian PrP.

## PrP displays antimicrobial activity

Key characteristics of AMPs, in addition to nucleic acid/membrane binding (see above), include (i) evolutionary conservation, (ii) induction by pathogen infection, and (iii) antimicrobial action via aggregation, features shared by PrP.

First, PrP is substantially conserved through evolution, with homologs in frogs and fish (Fig. 1B).

**Fig. 2:**
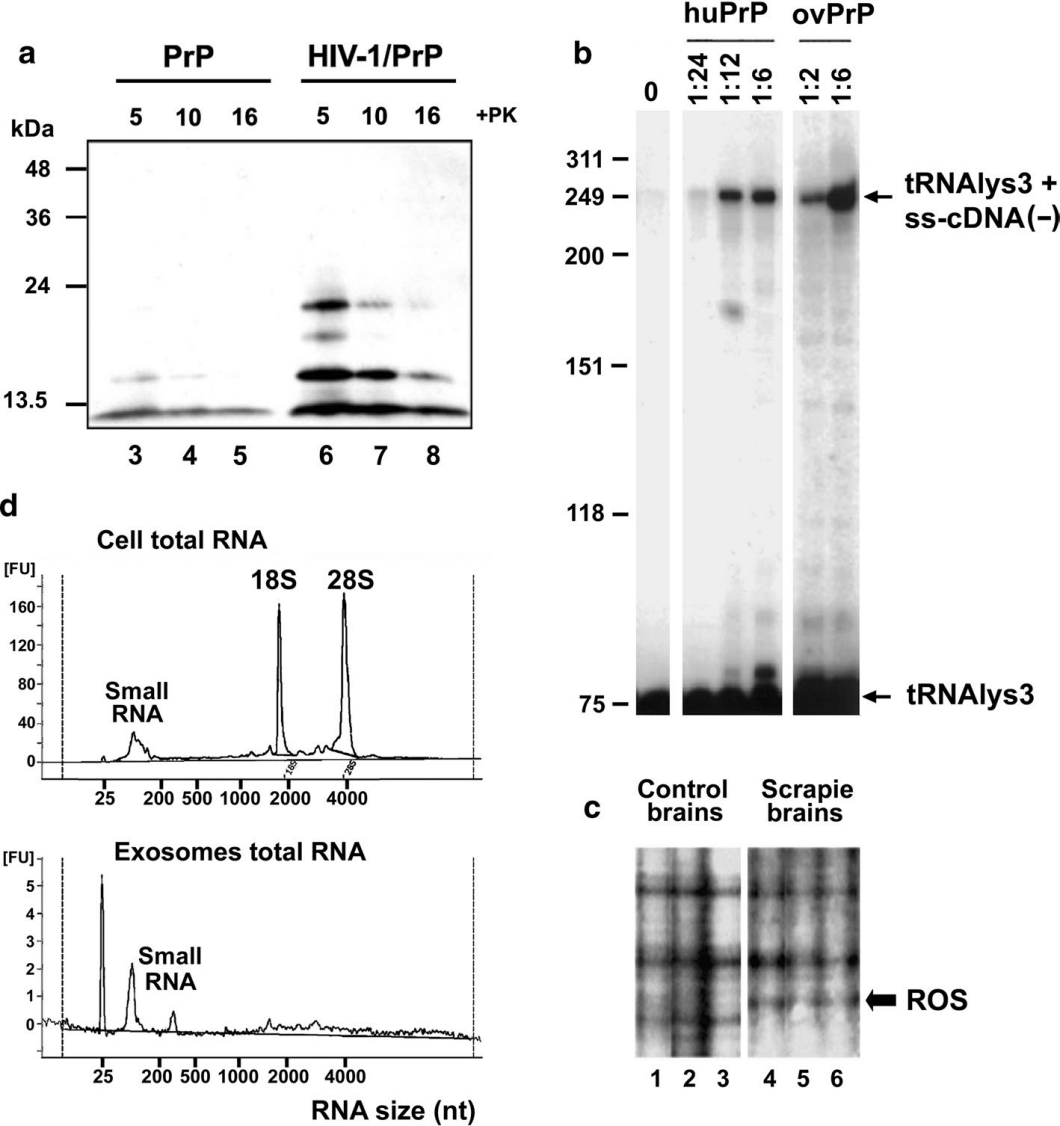
PrP interactions with retroviruses, retroelements, and exosomes. (A) Protease-resistant PrP aggregates in retrovirus infection. HIV-1 infection induces the formation of PrP forms that are resistant to proteinase K (PK, concentrations as indicated). Adapted, with permission, from reference [170]. (B) PrP chaperones the initiation of reverse transcription of HIV-1 RNA. A fragment of HIV-1 5’ RNA containing the tRNA primer-binding site was incubated with tRNA_Lys_^3^, HIV-1 reverse transcriptase, and dNTP in the presence or absence of human (hu) or ovine (ov) PrP. Almost no product is synthesized in the absence of PrP (first lane). Size markers, nt; ratios indicate the PrP dilution. Panel adapted, with permission, from Darlix and colleagues [116]. (C) Differential display (panel for illustration) of control and scrapie-infected brain led to the identification of ROS (RNA overexpressed in scrapie) sequences centrally including LINE elements and LINE targets. Lanes 1–3 and 4–6 are amplification products obtained using brain RNAs from independent control and scrapie-infected animals. Panel adapted, with permission, from reference [242]. (D) Size distribution of RNA in total cell extracts (above) and exosomes (below) from transmissible spongiform encephalopathy (TSE)-infected and control cells, illustrating RNA components as large as 300 nt, and possibly others in the > 1 kb range. Exosomes contain both PrP protein and TSE infectivity [217, 220], but deep sequencing indicates that they contain a select group of cellular nucleic acids, of which ~ 50% are retroelement RNAs [221]. Figure adapted, with permission, from reference [221]

Second, PrP expression is upregulated *in vitro* by infection with adenovirus 5 [156, 157], Epstein–Barr virus (EBV) [158], hepatitis C virus [159, 160], HIV-1 [161], *Helicobacter pylori* [162], and *Mycobacterium bovis* [163], as well as by murine leukemia virus (MuLV) and vesicular stomatitis virus [164]. *In vivo*, brain PrP is upregulated in HIV-1 infection as well as in simian immunodeficiency virus (SIV) encephalitis in macaques [165]. Of note, HIV is reported to induce PrP^Sc^-like PrP aggregation (Fig. 2A), which is also seen during infection with another retrovirus, caprine arthritis encephalitis virus [166].

Third, Schmidtchen and colleagues [167] were the first to report that PrP polypeptides display direct antimicrobial activity against Gram-negative and Gram-positive bacteria, as well as against the fungus *Candida parapsilosis*. The key region was mapped to the N-terminal domain, and studies on synthetic peptides confirmed the central role of region 1 [167]. In addition to bacteria and yeast, PrP is also known to restrict the proliferation of multiple DNA and RNA viruses, including adenovirus 5, coxsackievirus B3, HIV-1, and poliovirus (see reference [149] for review). In addition to aggregation induced on infection (see below), like other AMPs, PrP is also an immunomodulator (see reference [168] for review).

In sum, the data suggest that the primary ancestral function of PrP is as an antimicrobial defense protein. The presence of both long fibrils and condensed aggregates in TSE brain is consistent with the antimicrobial role of PrP, specifically in that this resembles the aggregation process inferred for Alzheimer disease (AD) Aβ peptide – extrusion of long filaments followed by condensation into dense aggregates that trap pathogens [140, 169]. Indeed, early researchers on TSE were struck by the resemblance between the deposits seen in TSE brain and those reported in AD [14, 15].

## Abnormal RNAs promote PrP refolding and aggregation: PrP^C^-to-PrP^Sc^ conversion as a sequestration mechanism

Nucleic acid binding is likely to be a central component of the antimicrobial repertoire of PrP. We underline two distinct mechanisms: (i) binding and (ii) sequestration. First, PrP can bind to HIV mRNA, which in turn blocks translation of the viral message, and native PrP inhibits HIV replication [170]. This activity has been confirmed for human, mouse, and hamster PrP [171] and is thus evolutionarily conserved.

The second mechanism involves aggregation. The propensity of AMPs to aggregate in response to pathogen ligands is generally accepted to be a major component of their defense activity [172, 173]. For PrP, the formation of the aggregated protease-resistant form is induced by infection (e.g., HIV-1 [170], Fig. 2A), and specific nucleic acids can trigger the conversion from PrP^C^ to PrP^Sc^ (see below) in which the protein refolds and subsequently aggregates. Aggregation may represent a sequestration mechanism that contributes to host defense [149], as it is generally for other AMPs such as Aβ [169]. For many AMPs, the trigger for aggregation is not known, but binding of nucleic acids to PrP can induce refolding of the molecule and generation of the aggregation-prone PrP^Sc^ form. The exact structural features remain unclear, but different nucleic acids differ enormously in their ability to catalyze this transition, as summarized below.

Binding of long (but not short) DNA can stimulate the conversion, and excess DNA, conversely, inhibits aggregation [105]. These effects are sequence-dependent. In an *in vitro* amplification system, poly(A) RNA was shown to be essential for the generation of PrP^Sc^. Although mammalian RNA preparations stimulated amplification of PrP^Sc^, RNA preparations from invertebrate species appeared not to do so ([76, 174]; reviewed in reference [175]), indicating that PrP recognizes specific features. Moreover, PrP molecules of different species (e.g., mouse versus hamster) appear to differ in their dependence on RNA for conversion to the PrP^Sc^ form [176].

Different RNAs have widely different binding affinities for PrP. RNAs with multiple double-stranded regions have been reported to bind most tightly [177, 178], and such highly structured RNAs promote the conversion of PrP (PrP^C^) to PrP^Sc^ [178]. Although the precise features that demarcate high-affinity binding to PrP have not been established, PrP is likely to be a sensor of non-Watson–Crick base pairs in double-stranded RNA [177], adjacent stem-loop structures and G4 quadruplexes (reviewed in reference [179]), and/or pseudoknots [180].

Notably, the binding of a single nucleic acid to two or more copies of PrP would bring different PrP molecules into close proximity, thereby promoting protein–protein interaction and aggregation. Abnormal RNAs triggering PrP^C^-to-PrP^Sc^ conversion are thus likely become entrapped in an insoluble aggregate, where they can no longer participate in cellular metabolism, and RNA sequestration is likely to contribute to the antimicrobial repertoire of PrP [149].

## PrP interactions with RNA and chaperoning of reverse transcription: implications for the nature of the TSE agent

### Pathogens exploit AMPs

Vertebrate AMPs and viruses have coexisted for at least 200 million years, and multiple viruses have co-opted AMPs to promote their own replication. For example, HIV-1 exploits the classical AMP LL-37 as well as Alzheimer Aβ to promote its own replication [181, 182], and there are several other examples [183–185]. The same is true of PrP, where HSV-1 has evolved an anti-PrP function, ICP34.5 (infected cell polypeptide 34.5 kDa), not only to evade PrP-mediated inactivation but also to exploit PrP to foster its own proliferation (reviewed in reference [149]). Another virus, hepatitis C virus, also exploits PrP to promote its own replication [186], as does MuLV [187]. This raises the possibility that an infectious agent, so far unknown, might exploit PrP in TSE. In the following, we focus on potential synergies with retroviruses and retroelements.

## PrP is a Gag-like protein that chaperones conversion of RNA to DNA by RT enzyme

A further dimension of PrP nucleic acid interactions was uncovered when it was observed that native PrP is capable of chaperoning the RT enzyme in retroviral cDNA synthesis assays [104, 116]. Briefly, after entry of the retroviral single-stranded RNA genome into the cell, RT-mediated synthesis of a complementary DNA strand is primed by an endogenous tRNA molecule. This involves the assembly of a macromolecular complex containing both the RNA genome template and a primer tRNA, a process that is normally promoted by the viral Gag nucleocapsid (NC) ‘chaperone’ protein. It was discovered that PrP is as effective as, or even more effective than HIV-1 NC in chaperoning RT-mediated cDNA synthesis [104, 116]. In the absence of a chaperone, almost no cDNA product is made, but the addition of either human or ovine PrP leads to a dramatic increase in the amount of cDNA (Fig. 2B). This has also been confirmed for feline immunodeficiency virus (FIV) [170]. The functional domain responsible for RT stimulatory activity is located within the N-terminal nucleic-acid-binding region of PrP [104] (Fig. 1). These findings indicate that the RNA-binding and chaperoning activities of PrP, a defense molecule that normally protects the host against virus infection, could potentially be subverted by retroviruses/retroelements to ensure their replication. PrP-mediated specific RT chaperoning has not yet been formally confirmed to take place *in vivo*, but PrP is necessary *in vivo* for HSV replication (which depends on retroelement activation), can promote MuLV proliferation [187, 188], colocalizes with both HIV-1 and MuLV Gag RT chaperone proteins [170, 187, 189], and is found in retrovirus particles [104, 116], suggesting that PrP is likely to contribute to the *in vivo* RT process.

## TSE and retroviruses

Synergistic interactions between TSE and retroviruses are well documented. In mouse NIH3T3 cells, which are poorly permissive for scrapie propagation, PrP^Sc^ production was not sustained following scrapie infection, but when the cells were coinfected with MuLV, there was a marked increase in both PrP^Sc^ levels and infectivity [189]. *In vivo*, higher brain titers of endogenous MuLV correlated with faster scrapie progression [190], and PrP boosted MuLV propagation [187], indicating that TSE and retrovirus infection act synergistically. Moreover, there is direct evidence for retrovirus mobilization in TSEs (Table 1A). TSE infection in multiple species is thus associated with endogenous retrovirus expression/proliferation.

**Table 1:** Retrovirus and retroelement mobilization in TSE

**Element**	**Findings**	**References**
**A. Retrovirus; host species**		
MuLV; mouse	TSE coinfection can promote MuLV proliferation	[187, 188]
Endogenous retroviruses (ERVs); mouse	ERV sequences constituted 2 of 22 specific markers upregulated in early-stage scrapie infection	[243]
Retrovirus; elk	A retroviral insertion site was a primary diagnostic DNA sequence marker in chronic wasting disease	[244]
ERVs; macaque	BSE infection upregulates a panel of ERVs	[245]
Retrovirus; human	Retrovirus-specific sequences in infectious fractions from CJD brain but not in controls	[246, 247]
Human ERVs (HERVs)	HERV detection rates were significantly increased in CJD samples; profiles were also distinctly different: 21 of 87 sporadic CJD samples, but none of 40 controls, were dual positive for HERV types W and L	[248]
**B. Retroelement; host species**		
Bov-tA; bovine	All infected cattle were positive for Bov-tA sequences (a short interspersed sequence mobilized by LINEs); only 5/845 healthy controls were positive	[249]
IAP-1; mouse	Scrapie infection in cultured mouse cells is associated with upregulation of IAP-1 retroelement RNA; ‘curing’ (i.e., removal of scrapie infection) of infected cells using pentosan polysulfate led to a remarkable (10^3^-fold) downregulation of IAP-1 RNA	[250]
LINE; hamster	The most abundant scrapie-only sequence in scrapie-infected hamster brain versus controls was a LINE1 family element. Other bands were RNA 7SL (SINE parent and common partner of LINE mobilization), and target sites for LINE-family elements that insert within 18S and 28S rDNA genes	[242]
7S RNA-related sequences; hamster	Hyperabundance of 7SK-hybridizing sequences in scrapie-infected brain	[251]

## TSE and retroelements

Retroviruses in the mammalian genome are outnumbered by endogenous retrovirus-like retroelements that replicate by an RT mechanism but lack an envelope glycoprotein and are thus incapable of generating conventional viral particles). These elements, including LINE family (long interspersed nuclear element) and related elements (such as short interspersed nuclear elements, SINES – B1/B2 elements in mice – highly structured Alu-like elements derived from cellular RNA 7SL), comprise up to 40% of the mammalian genome and are believed to have played, and continue to play, a crucial role in vertebrate genome evolution (reviewed in reference [191]). Some tissues, notably the brain, display active LINE/SINE transposition into adulthood [192, 193], and ongoing (physiological) mobilization of retroelements in the human brain is mainly of LINEs and SINES [194].

Importantly, new LINE integrations tend to take place into actively transcribed genes [195, 196], but element insertion typically leads to 5’ truncation and loss of the Gag-like chaperone activity encoded by the first open reading frame (ORF1) [197, 198]. Further mobilization may therefore become dependent on non-LINE RT chaperones such as PrP.

Thus, as summarized in Table 1B, in addition to retroviruses, there is direct evidence for retroelement mobilization in TSE infection in both rodents and cattle.

## Overlap with herpes virus biology: *Herpesviridae* members mobilize retroelements

As noted earlier, HSV-1 depends on PrP for its replication. This is relevant because members of the family *Herpesviridae*, including HSV1, cytomegalovirus (CMV), and EBV, activate the expression of human endogenous retroviruses (HERV)-K and HERV-W [199–206]. Both HSV1 [207–209] and gammaherpesvirus (MHV68 [210]) promote the expression of short interspersed nuclear elements (SINEs), short elements that depend on RT for mobilization, and SINE upregulation enhances herpesviral gene expression [210] via pathways that remain poorly understood. Further research will be necessary to determine whether interactions between PrP and SINE RNAs underlie the dependence of HSV-1 on PrP function.

## PrP transports nucleic acids

Retroelements such as LINEs are generally thought of as being wholly intracellular entities. Because (unlike retroviruses) they lack envelope proteins, it might be held that they cannot be taken up by cells, and, conversely, once inside the cell they have no mechanism for packaging and export from the cell. If so, this would rule them out as transmissible agents. However, the ability of AMPs such as PrP to bind to both membranes and nucleic acids has an unexpected consequence – nucleic acid delivery.

*Nucleic acid import.* The archetypical AMP LL-37 can bind to extracellular DNA plasmids and oligonucleotides and then transport them across the membrane into the cytosol and nucleus [138, 139]. Similar findings have been reported for PrP. Kocisko *et al.* expressed a fusion protein between PrP and GFP and studied binding and uptake of rhodamine-labeled ssDNA oligonucleotides. Initially, rhodamine fluorescence colocalized with GFP at the cell surface, but after 24 h, oligonucleotide fluorescence was concentrated in the perinuclear region; internalization was dependent on the fusion protein [211]. Magzoub *et al.* studied a fluorescein-conjugated N-terminal PrP peptide and reported a 100-fold increase in the internalization of ssDNA [212]. Equivalent findings were reported for a luciferase reporter plasmid, where a PrP peptide facilitated both uptake and luciferase gene expression. In addition, Yin *et al.* described experiments in which a PrP peptide construct internalized both dsDNA and ssDNA oligonucleotides. Expression of the plasmid reporter (YFP) was stimulated by at least two orders of magnitude by the PrP peptide [213]. This work shows that PrP can catalyze the uptake of extracellular nucleic acids into cells. Although it has been argued that PrP (and Aβ) uptake may take place via the laminin receptor [214], blockade of the receptor only reduced uptake by 20–55% [215, 216]; other receptors and/or direct membrane interactions are therefore likely to contribute to internalization.

*Nucleic acid export.* Cell disruption as a result of disease is one way in which intracellular nucleic acids can be released into the extracellular milieu. However, there is evidence for a more direct route. It has been known for many years that PrP associates with exosomes [217], small membrane-enclosed vesicles that are actively shed from the cell membrane of diverse cell types and contain cellular RNAs (reviewed in references [218, 219]). Exosomal PrP could thereby facilitate both nucleic acid binding and membrane interactions.

Importantly, exosomes secreted from scrapie-infected cells efficiently transmitted infection when inoculated into mice [217, 220]. Deep sequencing of RNAs present in vesicles released from cells infected with the human CJD-derived Fukuoka-1 TSE strain revealed that over 50% corresponded to retroviruses, LINES, and SINES [221] (Fig. 2D). Moreover, N-terminal epitopes of native PrP in infectious exosomes are masked against antibody recognition by an unknown ligand/modification [220], and it is possible that the PrP N-terminal region is tightly bound to some of these RNA species.

The specific association of infectivity, PrP protein, and retroelement sequences therefore suggests that PrP can act analogously to retroelement Gag proteins (analogs of LINE element ORF1 protein) in recruiting RNA genomes to membranes for export from the cell (e.g., [222, 223]).

## Nature of the natural TSE agent

Condensation of PrP^C^ into insoluble aggregates is, as with other AMPs, overtly a host response to entrap and inactivate the target pathogen (in this case, specific nucleic acids), but PrP^Sc^ formation only takes place late in infection (reviewed in reference [5]) – and sometimes not at all if there is mismatching between donor and recipient (e.g., first passages of BSE in mice [56–58, 60]), despite high titers of infectivity – raising the question of the molecular form of the infectious TSE agent before it is sequestered into PrP^Sc^ aggregates.

The most likely (natural) form of the transmissible agent is, arguably, an exosome-like phospholipid particle that also contains PrP and RNAs, notably retroelement RNAs or fragments thereof. This notion is based on the fact that PrP resembles the retroviral structural polyprotein Gag: both bind nucleic acids, interact with membranes, form aggregates in response to RNA binding, and have RNA chaperoning activities (i.e., fraying, unwinding, and annealing activities, matchmaking, and stimulation of RT activity by primer–template annealing and enzyme recruitment to the complex). Like Gag proteins, PrP can form liquid droplets upon binding to RNA in association with other RNA-binding proteins. Moreover, biophysical considerations argue that liquid droplets are precursors for the assembly of membrane-enclosed ribonucleoprotein complexes including endosomes/exosomes and retroviral particles. For example, lipid-associated PrP^Sc^ was reported to readily form liposomes [63]. However, the details need to be worked out.

Retrovirus production by budding proceeds via host-cell late endosomes and exosome pathways, and retrovirus particles and exosomes display many similarities. In addition to retroelement nucleic acids that are enriched in exosomes [221], PrP protein (as well as Gag proteins) is found in both retrovirus particles and exosomes [104, 116, 189, 217]. PrP is present on the outer surface, where it could plausibly promote membrane fusion, but is undoubtedly also within the particles – many forms of PrP lack the GPI membrane anchor, and PrP is recruited to particles even when the GPI anchor is missing [189].

TSE infectivity is found in exosomes [217, 220], and both the cellular (PrP^C^) and disease-related (PrP^Sc^) forms of PrP are present in exosomes as well as in retroviral (MoMLV and HIV) particles [104, 116, 189]. Indeed, GPI-linked proteins (such as PrP) are selectively recruited into both exosomes and retroviral particles (see above). As originally shown by Temin and Baltimore [224], RT is present in the interior of retroviral particles and can catalyze reverse transcription *in situ*, raising the possibility that retroelement-encoded RT enzyme may also be present in infectious exosomes (although this remains to be investigated). In support, Kato *et al.* list LINE1 (LINE1-type transposase domain-containing 1) as a component of RNA granule liquid droplets [225].

In sum, the PrP^Sc^ aggregates – a product of host defense – are unlikely to represent the form of the agent that transits between animals and cells *in vivo*. We suggest that subviral exosomal particles containing PrP and nucleic acid represent the infectious moiety in natural scrapie – with transmission via scratching posts or placenta.

## Is it PrP or the nucleic acid that causes pathology in TSE?

We have argued that PrP is a defense molecule that aggregates in response to specific RNAs but in some cases can provoke their RT copying and mobilization. However, like all AMPs, high concentrations of PrP are undoubtedly neurotoxic, particularly in their activated forms (such as Aβ peptide and PrP^Sc^), raising the question of whether it is the neurotoxic AMP or the microbe that causes the disease. For Aβ, the debate continues to rage, but the presence of extensive Aβ aggregates in the brain of healthy elderly individuals with no evidence of cognitive decline suggests that Aβ deposition has successfully immobilized the invader and is not itself the primary cause of disease [140]. The same line of argument may apply to TSE, because Yuan *et al.* [226] reported protease-resistant aggregates of PrP^Sc^-like material in normal human brain from individuals free of any neurological disorder (or *PRNP* mutations).

PrP^Sc^ can clearly be neurotoxic, but we argue that the neuropathology – which can take place in the absence of any PrP^Sc^ – is primarily caused by PrP-mediated retroelement mobilization, with widespread insertional mutagenesis and disruption of basic cellular metabolic processes (e.g., Alu retroelements are closely related to essential 7S RNAs that are involved in fundamental aspects of cell function such as protein secretion and translation).

## Discussion and conclusions: TSE as a retromobilization disease

In this synthesis we juxtapose new findings that were not available at the beginning of the prion era: first, that PrP is a nucleic-acid-binding antimicrobial protein that it similar to retroviral Gag proteins in its ability to trigger reverse transcription; second, that retroelement mobilization is widely seen in TSE disease; and third, that PrP can also mediate nucleic acid transport into and out of the cell.

**Fig. 3:**
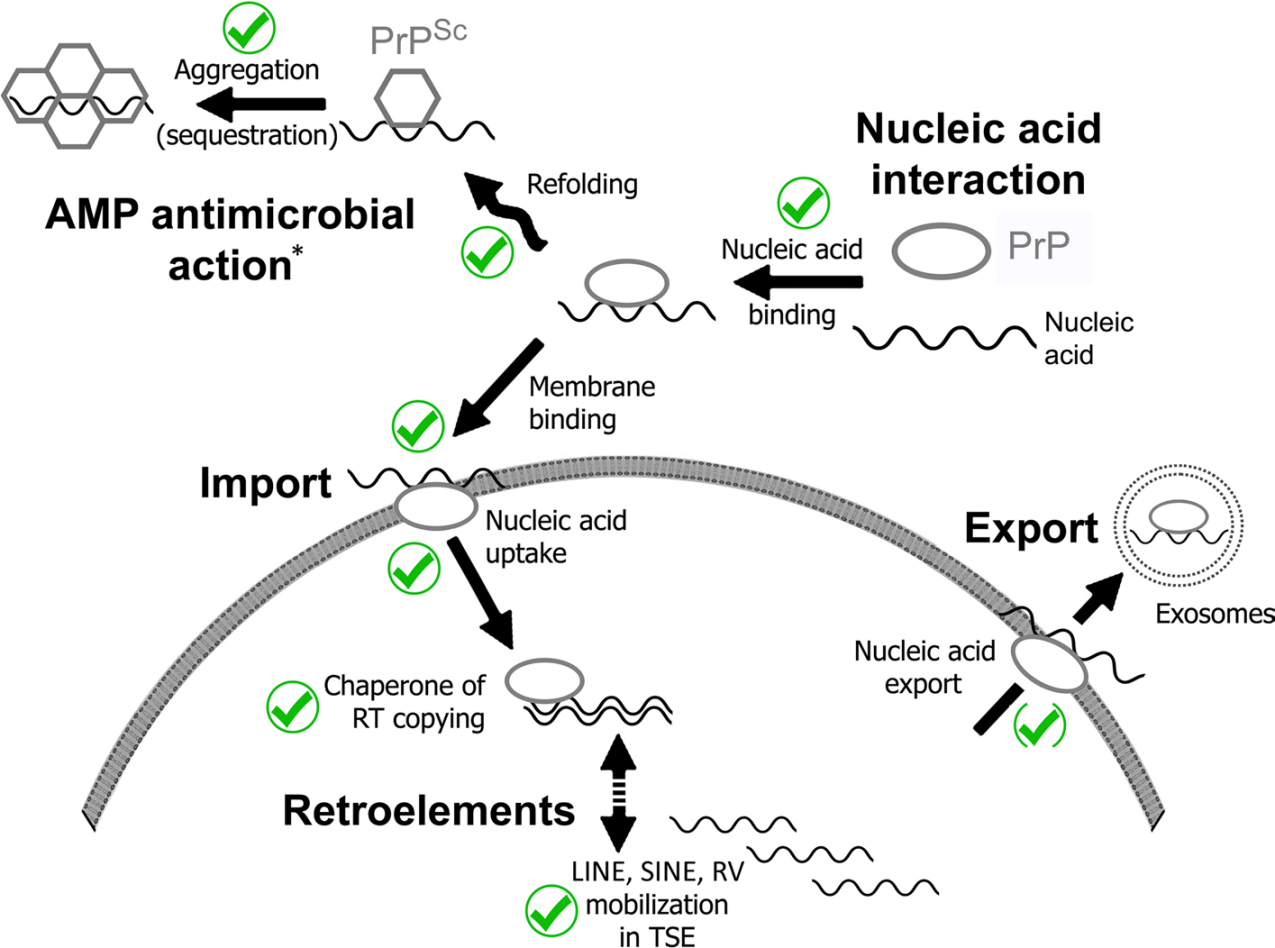
PrP promotes multiple steps in nucleic acid transport and retroelement mobilization. As reviewed in the text, PrP binds to nucleic acids and can (i) facilitate cellular uptake by membrane binding and/or (ii) undergo a conformation change in response to abnormal nucleic acids, which leads to aggregation as part of its AMP action (*the AMP activity of PrP may also involve membrane binding). PrP is a highly effective chaperone of cDNA synthesis by reverse transcriptase (RT), and the transmissible encephalopathies (TSEs) are characterized by upregulation/mobilization of retroelements, including long and short interspersed nuclear repeat elements (LINEs and SINES). TSE infectivity, PrP protein, and LINE nucleic acids are associated with membrane-enclosed exosomal vesicles that are shed from the cell surface. indicates confirmed steps. Abbreviations: AMP, antimicrobial peptide; RV, retrovirus

To explain the 30 or more strains of TSE, a strong case can now be made that a second element – retroelement nucleic acid – bound to PrP constitutes the second component. We propose that a retroelement nucleic acid bound to PrP constitutes the infectious agent, triggering uncontrolled retroelement mobilization in the recipient and onward transmission to adjacent cells (Fig. 3). This analysis suggests that strain characteristics are determined by the identity of the retroelement nucleic acid(s) bound to PrP.

There is a precedent for retrotransposition disease – hybrid dysgenesis in *Drosophila*. When a transposition-repressed genome is crossed into a permissive line, derepression takes place – leading to massive mobilization of both non-RT and RT elements, including classic LINE elements [227, 228], causing widespread insertional mutagenesis and cell toxicity.

Unexpectedly, retroelements (like retroviruses) can also be transmitted between hosts. This has been amply documented for mobile elements in insects and plants (not reviewed), but can also take place in vertebrates. Ruminants (*Bos* and *Ovis* spp.) recently (~ 40 million years ago) acquired a specific LINE, BovB, from snakes and lizards, and it has been suggested that biting insects may have been the vector [229, 230]. Classical L1 elements can also be transferred between species [231].

In TSE, the simplest interpretation is that a retroelement/endogenous retrovirus RNA genome, or a subfragment thereof, is brought into the cell by PrP, and PrP chaperone activity then promotes its copying into DNA and genomic insertion. A retroelement subfragment might suffice in some cases, acting as a primer for PrP-stimulated reverse transcription of an endogenous element and subsequent mobilization. The disease-causing properties of a given inoculum would then crucially depend on the extent of matching between the incoming nucleic acid and host-encoded elements. This could explain the species barrier that is observed with some TSE agents (and also mutational changes as a consequence of mismatching).

PrP (and potentially other Gag-like nucleic acid chaperones) clearly plays a vital role in this process because free nucleic acid from TSE brain is not infectious (e.g., [66, 88]; note our earlier caveat regarding an unusual brain-enriched RNase), but one observation remains to be explained – that disease-associated forms of PrP may, at low frequency, alone establish infection, a process that probably requires a stochastic event taking place in the host cell. What might this event be?

We envisage two non-exclusive scenarios. First, a chance tripartite encounter between the PrP inoculum, an endogenous retroelement mRNA (or DNA), and a cellular RNA (or fragment) capable of acting as a primer could set up mobilization of the retroelement, leading to disease. It is of note that LINE-type retroelements typically lack the upstream ORF encoding the crucial Gag-like RT chaperone protein and thus cannot mobilize. Exogenous PrP (possibly refolded into an alternative conformation) could therefore catalyze *de novo* mobilization of otherwise silent elements. Second, sporadic mutation in a endogenous retroelement RNA (or gene) could lead to an altered RNA conformation that is efficiently mobilized by PrP.

Under this interpretation, the appearance of spontaneous disease in familial TSEs, such as CJD and GSS, which harbor disease-triggering mutations in PrP, could thus result from PrP-mediated hyperactivation of endogenous elements. In support, cells containing PrP mutated to contain the P102L GSS allele (P101L in mouse PrP) displayed higher MuLV titers *in vitro* and increased MuLV plaque size [187]. Although this remains to be independently confirmed, this finding suggests that familial TSE mutant PrP can upregulate the proliferation of an endogenous element.

Looking wider, abnormal retroelement mobilization has also been reported in neuropsychiatric diseases including Alzheimer disease [232–234], of note because PrP interacts with Alzheimer Aβ (see text and reference [149] for review), leading to the inference that PrP chaperone activity may also contribute to pathology in other diseases.

In sum, the data argue for an intimate association between PrP and nucleic acids that could finally explain the different strains of TSE agent. More than a decade ago, we were, in the words of Silva *et al.*, ‘halfway there’ towards the identification of the specific nucleic acid(s) involved in TSE [235]. We surmise that we are today three-quarters of the way towards that goal. This is an important goal because it raises pragmatic issues relating to TSE diagnosis, disinfection, and potential therapeutics. However, advocates of the protein-only hypothesis will rightly demand a formal proof before admitting any modification to the theory. Only time (and further experiment) will tell. Although deep sequencing of nucleic acids from TSE versus control will hopefully cast light, testing the infectivity of specific nucleic acids *in vivo* in conjunction with purified PrP will be necessary to resolve the matter, but this requires pathogen containment facilities and is not a trivial undertaking. To conclude, as Moira Bruce observed a quarter of a century ago, ‘The issue will remain controversial until there is a direct identification of the informational molecule of the agent and the variations in it which lead to phenotypic diversity’ [34].

## References

[1] Weissmann C (1991). A ‘unified theory’ of prion propagation. Nature.

[2] Bruce ME, Dickinson AG (1987). Biological evidence that scrapie agent has an independent genome. J Gen Virol.

[3] Silva JL, Lima LM, Foguel D, Cordeiro Y (2008). Intriguing nucleic-acid-binding features of mammalian prion protein. Trends Biochem Sci.

[4] Manuelidis L (2003). Transmissible encephalopathies: speculations and realities. Viral Immunol.

[5] Manuelidis L (2013). Infectious particles, stress, and induced prion amyloids: a unifying perspective. Virulence.

[6] Cleeland B (2009) The Bovine Spongiform Encephalopathy (BSE) Epidemic in the United Kingdom. International Risk Governance Council

[7] Cuillé J, Chelle PL (1936). La maladie dite tremblante du mouton est-elle inoculable?. C R Acad Sci.

[8] Barrairon E (1989). La découverte par Cuillé et Chelle des ‘maladies virales lentes’ a l’Ecole Vétérinaire de Toulouse dans les années 30: un témoignage a l’ombre des ‘inventeurs’. Bull Acad Vét France.

[9] Gibbs CJ, Gajdusek DC, Asher DM, Alpers MP, Beck E, Daniel PM, Matthews WB (1968). Creutzfeldt-Jakob disease (spongiform encephalopathy): transmission to the chimpanzee. Science.

[10] Manuelidis L, Chakrabarty T, Miyazawa K, Nduom NA, Emmerling K (2009). The kuru infectious agent is a unique geographic isolate distinct from Creutzfeldt-Jakob disease and scrapie agents. Proc Natl Acad Sci USA.

[11] Plummer PJ (1946). Scrapie—a disease of sheep: a review of the literature. Can J Comp Med Vet Sci.

[12] Field EJ, Peat A (1969). Structural changes in scrapie affected brain. Biochem J.

[13] Fraser H, Bruce M (1973). Argyrophilic plaques in mice inoculated with scrapie from particular sources. Lancet.

[14] Wisniewski HM, Bruce ME, Fraser H (1975). Infectious etiology of neuritic (senile) plaques in mice. Science.

[15] Merz PA, Somerville RA, Wisniewski HM, Iqbal K (1981). Abnormal fibrils from scrapie-infected brain. Acta Neuropathol.

[16] Bolton DC, McKinley MP, Prusiner SB (1982). Identification of a protein that purifies with the scrapie prion. Science.

[17] Diringer H, Gelderblom H, Hilmert H, Ozel M, Edelbluth C, Kimberlin RH (1983). Scrapie infectivity, fibrils and low molecular weight protein. Nature.

[18] McKinley MP, Bolton DC, Prusiner SB (1983). A protease-resistant protein is a structural component of the scrapie prion. Cell.

[19] Prusiner SB, McKinley MP, Bowman KA, Bolton DC, Bendheim PE, Groth DF, Glenner GG (1983). Scrapie prions aggregate to form amyloid-like birefringent rods. Cell.

[20] Oesch B, Westaway D, Walchli M, McKinley MP, Kent SB, Aebersold R, Barry RA, Tempst P, Teplow DB, Hood LE (1985). A cellular gene encodes scrapie PrP 27–30 protein. Cell.

[21] Wulf MA, Senatore A, Aguzzi A (2017). The biological function of the cellular prion protein: an update. BMC Biol.

[22] Castle AR, Gill AC (2017). Physiological functions of the cellular prion protein. Front Mol Biosci.

[23] Sakudo A, Onodera T (2014). Prion protein (PrP) gene-knockout cell lines: insight into functions of the PrP. Front Cell Dev Biol.

[24] Prusiner SB (1982). Novel proteinaceous infectious particles cause scrapie. Science.

[25] Prusiner SB (1998). Prions. Proc Natl Acad Sci USA.

[26] Weissmann C (2004). The state of the prion. Nat Rev Microbiol.

[27] Aguzzi A, Calella AM (2009). Prions: protein aggregation and infectious diseases. Physiol Rev.

[28] Rohwer RG (1984). Scrapie infectious agent is virus-like in size and susceptibility to inactivation. Nature.

[29] Alper T, Cramp WA, Haig DA, Clarke MC (1967). Does the agent of scrapie replicate without nucleic acid?. Nature.

[30] Griffith JS (1967). Self-replication and scrapie. Nature.

[31] Pattison IH, Jones KM (1967). The possible nature of the transmissible agent of scrapie. Vet Rec.

[32] Prusiner SB, Gabizon R, McKinley MP (1987). On the biology of prions. Acta Neuropathol.

[33] Fraser H, Dickinson AG (1973). Scrapie in mice. Agent-strain differences in the distribution and intensity of grey matter vacuolation. J Comp Pathol.

[34] Bruce ME (1993). Scrapie strain variation and mutation. Br Med Bull.

[35] Masujin K, Okada H, Miyazawa K, Matsuura Y, Imamura M, Iwamaru Y, Murayama Y, Yokoyama T (2016). Emergence of a novel bovine spongiform encephalopathy (BSE) prion from an atypical H-type BSE. Sci Rep.

[36] Bessen RA, Marsh RF (1992). Identification of two biologically distinct strains of transmissible mink encephalopathy in hamsters. J Gen Virol.

[37] Perrott MR, Sigurdson CJ, Mason GL, Hoover EA (2012). Evidence for distinct chronic wasting disease (CWD) strains in experimental CWD in ferrets. J Gen Virol.

[38] Galeno R, Di Bari MA, Nonno R, Cardone F, Sbriccoli M, Graziano S, Ingrosso L, Fiorini M, Valanzano A, Pasini G, Poleggi A, Vinci R, Ladogana A, Puopolo M, Monaco S, Agrimi U, Zanusso G, Pocchiari M (2017). Prion strain characterization of a novel subtype of Creutzfeldt-Jakob disease. J Virol.

[39] Dickinson AG, Fraser H, Meikle VM, Outram GW (1972). Competition between different scrapie agents in mice. Nat New Biol.

[40] Dickinson AG, Fraser H, McConnell I, Outram GW, Sales DI, Taylor DM (1975). Extraneural competition between different scrapie agents leading to loss of infectivity. Nature.

[41] Manuelidis L (1998). Vaccination with an attenuated Creutzfeldt-Jakob disease strain prevents expression of a virulent agent. Proc Natl Acad Sci USA.

[42] Nishida N, Katamine S, Manuelidis L (2005). Reciprocal interference between specific CJD and scrapie agents in neural cell cultures. Science.

[43] Henle W, Henle G (1943). Interference of inactive virus with the propagation of virus of influenza. Science.

[44] Stauffer Thompson KA, Rempala GA, Yin J (2009). Multiple-hit inhibition of infection by defective interfering particles. J Gen Virol.

[45] Welsh RM, Lampert PW, Oldstone MB (1977). Prevention of virus-induced cerebellar diseases by defective-interfering lymphocytic choriomeningitis virus. J Infect Dis.

[46] Dickinson AG, Outram GW, Prusiner SB, Hadlow WJ (1979). The scrapie replication-site hypothesis and its implications for pathogenesis. Slow transmissible diseases of the nervous system.

[47] Mays CE, Kim C, Haldiman T, van der Merwe J, Lau A, Yang J, Grams J, Di Bari MA, Nonno R, Telling GC, Kong Q, Langeveld J, McKenzie D, Westaway D, Safar JG (2014). Prion disease tempo determined by host-dependent substrate reduction. J Clin Invest.

[48] Mays CE, van der Merwe J, Kim C, Haldiman T, McKenzie D, Safar JG, Westaway D (2015). Prion infectivity plateaus and conversion to symptomatic disease originate from falling precursor levels and increased levels of oligomeric PrPSc species. J Virol.

[49] Baxa U, Cassese T, Kajava AV, Steven AC (2006). Structure, function, and amyloidogenesis of fungal prions: filament polymorphism and prion variants. Adv Protein Chem.

[50] Zambrano R, Conchillo-Sole O, Iglesias V, Illa R, Rousseau F, Schymkowitz J, Sabate R, Daura X, Ventura S (2015). PrionW: a server to identify proteins containing glutamine/asparagine rich prion-like domains and their amyloid cores. Nucleic Acids Res.

[51] Sabate R, Rousseau F, Schymkowitz J, Ventura S (2015). What makes a protein sequence a prion?. PLoS Comput Biol.

[52] Peretz D, Williamson RA, Legname G, Matsunaga Y, Vergara J, Burton DR, DeArmond SJ, Prusiner SB, Scott MR (2002). A change in the conformation of prions accompanies the emergence of a new prion strain. Neuron.

[53] Legname G, Nguyen HO, Baskakov IV, Cohen FE, DeArmond SJ, Prusiner SB (2005). Strain-specified characteristics of mouse synthetic prions. Proc Natl Acad Sci USA.

[54] Collinge J, Clarke AR (2007). A general model of prion strains and their pathogenicity. Science.

[55] Silveira JR, Raymond GJ, Hughson AG, Race RE, Sim VL, Hayes SF, Caughey B (2005). The most infectious prion protein particles. Nature.

[56] Lasmezas CI, Deslys JP, Robain O, Jaegly A, Beringue V, Peyrin JM, Fournier JG, Hauw JJ, Rossier J, Dormont D (1997). Transmission of the BSE agent to mice in the absence of detectable abnormal prion protein. Science.

[57] Barron RM, Campbell SL, King D, Bellon A, Chapman KE, Williamson RA, Manson JC (2007). High titers of transmissible spongiform encephalopathy infectivity associated with extremely low levels of PrPSc in vivo. J Biol Chem.

[58] Balkema-Buschmann A, Eiden M, Hoffmann C, Kaatz M, Ziegler U, Keller M, Groschup MH (2011). BSE infectivity in the absence of detectable PrP(Sc) accumulation in the tongue and nasal mucosa of terminally diseased cattle. J Gen Virol.

[59] Lewis V, Haigh CL, Masters CL, Hill AF, Lawson VA, Collins SJ (2012). Prion subcellular fractionation reveals infectivity spectrum, with a high titre-low PrPres level disparity. Mol Neurodegener.

[60] Dobie K, Barron R (2013). Dissociation between transmissible spongiform encephalopathy (TSE) infectivity and proteinase K-resistant PrP(Sc) levels in peripheral tissue from a murine transgenic model of TSE disease. J Virol.

[61] Hill AF, Joiner S, Linehan J, Desbruslais M, Lantos PL, Collinge J (2001). Species-barrier-independent prion replication in apparently resistant species. Proc Natl Acad Sci USA.

[62] Bolton DC, Bendheim PE, Marmorstein AD, Potempska A (1987). Isolation and structural studies of the intact scrapie agent protein. Arch Biochem Biophys.

[63] Safar J, Wang W, Padgett MP, Ceroni M, Piccardo P, Zopf D, Gajdusek DC, Gibbs CJ (1990). Molecular mass, biochemical composition, and physicochemical behavior of the infectious form of the scrapie precursor protein monomer. Proc Natl Acad Sci USA.

[64] Wenborn A, Terry C, Gros N, Joiner S, D’Castro L, Panico S, Sells J, Cronier S, Linehan JM, Brandner S, Saibil HR, Collinge J, Wadsworth JD (2015). A novel and rapid method for obtaining high titre intact prion strains from mammalian brain. Sci Rep.

[65] Hill AF, Antoniou M, Collinge J (1999). Protease-resistant prion protein produced in vitro lacks detectable infectivity. J Gen Virol.

[66] Simoneau S, Thomzig A, Ruchoux MM, Vignier N, Daus ML, Poleggi A, Lebon P, Freire S, Durand V, Graziano S, Galeno R, Cardone F, Comoy E, Pocchiari M, Beekes M, Deslys JP, Fournier JG (2015). Synthetic scrapie infectivity: interaction between recombinant PrP and scrapie brain-derived RNA. Virulence.

[67] Schmidt C, Fizet J, Properzi F, Batchelor M, Sandberg MK, Edgeworth JA, Afran L, Ho S, Badhan A, Klier S, Linehan JM, Brandner S, Hosszu LL, Tattum MH, Jat P, Clarke AR, Klohn PC, Wadsworth JD, Jackson GS, Collinge J (2015). A systematic investigation of production of synthetic prions from recombinant prion protein. Open Biol.

[68] Legname G, Baskakov IV, Nguyen HO, Riesner D, Cohen FE, DeArmond SJ, Prusiner SB (2004). Synthetic mammalian prions. Science.

[69] Wang F, Wang X, Yuan CG, Ma J (2010). Generating a prion with bacterially expressed recombinant prion protein. Science.

[70] Wang F, Wang X, Ma J (2011). Conversion of bacterially expressed recombinant prion protein. Methods.

[71] Timmes AG, Moore RA, Fischer ER, Priola SA (2013). Recombinant prion protein refolded with lipid and RNA has the biochemical hallmarks of a prion but lacks in vivo infectivity. PLoS ONE.

[72] Bieschke J, Weber P, Sarafoff N, Beekes M, Giese A, Kretzschmar H (2004). Autocatalytic self-propagation of misfolded prion protein. Proc Natl Acad Sci USA.

[73] Castilla J, Saa P, Hetz C, Soto C (2005). In vitro generation of infectious scrapie prions. Cell.

[74] Weber P, Giese A, Piening N, Mitteregger G, Thomzig A, Beekes M, Kretzschmar HA (2007). Generation of genuine prion infectivity by serial PMCA. Vet Microbiol.

[75] Wang X, McGovern G, Zhang Y, Wang F, Zha L, Jeffrey M, Ma J (2015). Intraperitoneal infection of wild-type mice with synthetically generated mammalianprion. PLoS Pathog.

[76] Deleault NR, Harris BT, Rees JR, Supattapone S (2007). Formation of native prions from minimal components in vitro. Proc Natl Acad Sci USA.

[77] Deleault NR, Walsh DJ, Piro JR, Wang F, Wang X, Ma J, Rees JR, Supattapone S (2012). Cofactor molecules maintain infectious conformation and restrict strain properties in purified prions. Proc Natl Acad Sci USA.

[78] Elezgarai SR, Fernandez-Borges N, Erana H, Sevillano AM, Charco JM, Harrathi C, Saa P, Gil D, Kong Q, Requena JR, Andreoletti O, Castilla J (2017). Generation of a new infectious recombinant prion: a model to understand Gerstmann-Straussler-Scheinker syndrome. Sci Rep.

[79] Wang F, Wang X, Abskharon R, Ma J (2018). Prion infectivity is encoded exclusively within the structure of proteinase K-resistant fragments of synthetically generated recombinant PrP(Sc). Acta Neuropathol Commun.

[80] Burke CM, Walsh DJ, Steele AD, Agrimi U, Di Bari MA, Watts JC, Supattapone S (2019). Full restoration of specific infectivity and strain properties from pure mammalian prion protein. PLoS Pathog.

[81] Noble GP, Wang DW, Walsh DJ, Barone JR, Miller MB, Nishina KA, Li S, Supattapone S (2015). A structural and functional comparison between infectious and non-infectious autocatalytic recombinant PrP conformers. PLoS Pathog.

[82] Lessig J, Fuchs B (2010). HOCl-mediated glycerophosphocholine and glycerophosphoethanolamine generation from plasmalogens in phospholipid mixtures. Lipids.

[83] Hoover CE, Davenport KA, Henderson DM, Zabel MD, Hoover EA (2017) Endogenous brain lipids inhibit prion amyloid formation in vitro. J Virol 91 10.1128/JVI.02162-16 PMC5391467 28202758

[84] Konold T, Hawkins SA, Thurston LC, Maddison BC, Gough KC, Duarte A, Simmons HA (2015). Objects in contact with classical scrapie sheep act as a reservoir for scrapie transmission. Front Vet Sci.

[85] Zabel Mark D., Reid Crystal (2015). A brief history of prions. Pathogens and Disease.

[86] Diener TO, McKinley MP, Prusiner SB (1982). Viroids and prions. Proc Natl Acad Sci USA.

[87] Safar JG, Kellings K, Serban A, Groth D, Cleaver JE, Prusiner SB, Riesner D (2005). Search for a prion-specific nucleic acid. J Virol.

[88] Hunter GD, Collis SC, Millson GC, Kimberlin RH (1976). Search for scrapie-specific RNA and attempts to detect an infectious DNA or RNA. J Gen Virol.

[89] Eller CH, Lomax JE, Raines RT (2014). Bovine brain ribonuclease is the functional homolog of human ribonuclease 1. J Biol Chem.

[90] Botsios S, Manuelidis L (2016). CJD and scrapie require agent-associated nucleic acids for infection. J Cell Biochem.

[91] Miyazawa K, Emmerling K, Manuelidis L (2011). High CJD infectivity remains after prion protein is destroyed. J Cell Biochem.

[92] Kipkorir T, Tittman S, Botsios S, Manuelidis L (2014). Highly infectious CJD particles lack prion protein but contain many viral-linked peptides by LC–MS/MS. J Cell Biochem.

[93] Lathe R (1985). Synthetic oligonucleotide probes deduced from amino acid sequence data. Theoretical and practical considerations. J Mol Biol.

[94] Simoneau S, Ruchoux MM, Vignier N, Lebon P, Freire S, Comoy E, Deslys JP, Fournier JG (2009) Small critical RNAs in the scrapie agent. Nat Proc http://hdl.handle.net/10101/npre.2009.3344.1

[95] Calabretta S, Richard S (2015). Emerging roles of disordered sequences in RNA-binding proteins. Trends Biochem Sci.

[96] Wang C, Uversky VN, Kurgan L (2016). Disordered nucleiome: abundance of intrinsic disorder in the DNA- and RNA-binding proteins in 1121 species from Eukaryota, Bacteria and Archaea. Proteomics.

[97] Caughey BW, Dong A, Bhat KS, Ernst D, Hayes SF, Caughey WS (1991). Secondary structure analysis of the scrapie-associated protein PrP 27-30 in water by infrared spectroscopy. Biochemistry.

[98] Pan KM, Baldwin M, Nguyen J, Gasset M, Serban A, Groth D, Mehlhorn I, Huang Z, Fletterick RJ, Cohen FE (1993). Conversion of alpha-helices into beta-sheets features in the formation of the scrapie prion proteins. Proc Natl Acad Sci USA.

[99] Premzl M, Gready JE, Jermiin LS, Simonic T, Marshall Graves JA (2004). Evolution of vertebrate genes related to prion and Shadoo proteins - clues from comparative genomic analysis. Mol Biol Evol.

[100] Rivera-Milla E, Oidtmann B, Panagiotidis CH, Baier M, Sklaviadis T, Hoffmann R, Zhou Y, Solis GP, Stuermer CA, Malaga-Trillo E (2006). Disparate evolution of prion protein domains and the distinct origin of Doppel- and prion-related loci revealed by fish-to-mammal comparisons. FASEB J.

[101] Ciric D, Rezaei H (2015). Biochemical insight into the prion protein family. Front Cell Dev Biol.

[102] Corley SM, Gready JE (2008). Identification of the RGG box motif in Shadoo: RNA-binding and signaling roles?. Bioinform Biol Insights.

[103] Lau A, Mays CE, Genovesi S, Westaway D (2012). RGG repeats of PrP-like Shadoo protein bind nucleic acids. Biochemistry.

[104] Gabus C, Derrington E, Leblanc P, Chnaiderman J, Dormont D, Swietnicki W, Morillas M, Surewicz WK, Marc D, Nandi P, Darlix JL (2001). The prion protein has RNA binding and chaperoning properties characteristic of nucleocapsid protein NCP7 of HIV-1. J Biol Chem.

[105] Cordeiro Y, Machado F, Juliano L, Juliano MA, Brentani RR, Foguel D, Silva JL (2001). DNA converts cellular prion protein into the beta-sheet conformation and inhibits prion peptide aggregation. J Biol Chem.

[106] Gomes MP, Cordeiro Y, Silva JL (2008). The peculiar interaction between mammalian prion protein and RNA. Prion.

[107] Silva JL, Cordeiro Y (2016). The ‘Jekyll and Hyde’ actions of nucleic acids on the prion-like aggregation of proteins. J Biol Chem.

[108] Zou WQ, Zheng J, Gray DM, Gambetti P, Chen SG (2004). Antibody to DNA detects scrapie but not normal prion protein. Proc Natl Acad Sci USA.

[109] Knaus KJ, Morillas M, Swietnicki W, Malone M, Surewicz WK, Yee VC (2001). Crystal structure of the human prion protein reveals a mechanism for oligomerization. Nat Struct Biol.

[110] Satoh J, Obayashi S, Misawa T, Sumiyoshi K, Oosumi K, Tabunoki H (2009). Protein microarray analysis identifies human cellular prion protein interactors. Neuropathol Appl Neurobiol.

[111] Fischer M, Rulicke T, Raeber A, Sailer A, Moser M, Oesch B, Brandner S, Aguzzi A, Weissmann C (1996). Prion protein (PrP) with amino-proximal deletions restoring susceptibility of PrP knockout mice to scrapie. EMBO J.

[112] Supattapone S, Bosque P, Muramoto T, Wille H, Aagaard C, Peretz D, Nguyen HO, Heinrich C, Torchia M, Safar J, Cohen FE, DeArmond SJ, Prusiner SB, Scott M (1999). Prion protein of 106 residues creates an artifical transmission barrier for prion replication in transgenic mice. Cell.

[113] Weissmann C, Flechsig E (2003). PrP knock-out and PrP transgenic mice in prion research. Br Med Bull.

[114] Nandi PK, Leclerc E (1999). Polymerization of murine recombinant prion protein in nucleic acid solution. Arch Virol.

[115] Alred EJ, Nguyen M, Martin M, Hansmann UHE (2017). Molecular dynamics simulations of early steps in RNA-mediated conversion of prions. Protein Sci.

[116] Gabus C, Auxilien S, Pechoux C, Dormont D, Swietnicki W, Morillas M, Surewicz W, Nandi P, Darlix JL (2001). The prion protein has DNA strand transfer properties similar to retroviral nucleocapsid protein. J Mol Biol.

[117] Safar J, Wille H, Itri V, Groth D, Serban H, Torchia M, Cohen FE, Prusiner SB (1998). Eight prion strains have PrP(Sc) molecules with different conformations. Nat Med.

[118] Tzaban S, Friedlander G, Schonberger O, Horonchik L, Yedidia Y, Shaked G, Gabizon R, Taraboulos A (2002). Protease-sensitive scrapie prion protein in aggregates of heterogeneous sizes. Biochemistry.

[119] Sajnani G, Pastrana MA, Dynin I, Onisko B, Requena JR (2008). Scrapie prion protein structural constraints obtained by limited proteolysis and mass spectrometry. J Mol Biol.

[120] Turk E, Teplow DB, Hood LE, Prusiner SB (1988). Purification and properties of the cellular and scrapie hamster prion proteins. Eur J Biochem.

[121] Sajnani G, Silva CJ, Ramos A, Pastrana MA, Onisko BC, Erickson ML, Antaki EM, Dynin I, Vazquez-Fernandez E, Sigurdson CJ, Carter JM, Requena JR (2012). PK-sensitive PrP is infectious and shares basic structural features with PK-resistant PrP. PLoS Pathog.

[122] Cohen FE, Prusiner SB (1998). Pathologic conformations of prion proteins. Annu Rev Biochem.

[123] Lin Y, Protter DS, Rosen MK, Parker R (2015). Formation and maturation of phase-separated liquid droplets by RNA-binding proteins. Mol Cell.

[124] Drino A, Schaefer MR (2018). RNAs, phase separation, and membrane-less organelles: are post-transcriptional modifications modulating organelle dynamics?. BioEssays.

[125] Fonin AV, Darling AL, Kuznetsova IM, Turoverov KK, Uversky VN (2018). Intrinsically disordered proteins in crowded milieu: when chaos prevails within the cellular gumbo. Cell Mol Life Sci.

[126] Uversky VN (2019). Supramolecular fuzziness of intracellular liquid droplets: liquid–liquid phase transitions, membrane-less organelles, and intrinsic disorder. Molecules.

[127] Kostylev MA, Tuttle MD, Lee S, Klein LE, Takahashi H, Cox TO, Gunther EC, Zilm KW, Strittmatter SM (2018). Liquid and hydrogel phases of PrP(C) linked to conformation shifts and triggered by Alzheimer’s amyloid-beta oligomers. Mol Cell.

[128] Parchi P, Zou W, Wang W, Brown P, Capellari S, Ghetti B, Kopp N, Schulz-Schaeffer WJ, Kretzschmar HA, Head MW, Ironside JW, Gambetti P, Chen SG (2000). Genetic influence on the structural variations of the abnormal prion protein. Proc Natl Acad Sci USA.

[129] Silva CJ (2014). Applying the tools of chemistry (mass spectrometry and covalent modification by small molecule reagents) to the detection of prions and the study of their structure. Prion.

[130] Peretz D, Williamson RA, Matsunaga Y, Serban H, Pinilla C, Bastidas RB, Rozenshteyn R, James TL, Houghten RA, Cohen FE, Prusiner SB, Burton DR (1997). A conformational transition at the N terminus of the prion protein features in formation of the scrapie isoform. J Mol Biol.

[131] Khalili-Shirazi A, Summers L, Linehan J, Mallinson G, Anstee D, Hawke S, Jackson GS, Collinge J (2005). PrP glycoforms are associated in a strain-specific ratio in native PrPSc. J Gen Virol.

[132] Gielbert A, Thorne JK, Plater JM, Thorne L, Griffiths PC, Simmons MM, Cassar CA (2018). Molecular characterisation of atypical BSE prions by mass spectrometry and changes following transmission to sheep and transgenic mouse models. PLoS One.

[133] Howells LC, Anderson S, Coldham NG, Sauer MJ (2008). Transmissible spongiform encephalopathy strain-associated diversity of N-terminal proteinase K cleavage sites of PrP(Sc) from scrapie-infected and bovine spongiform encephalopathy-infected mice. Biomarkers.

[134] Zasloff M (2002). Antimicrobial peptides of multicellular organisms. Nature.

[135] Brogden KA (2005). Antimicrobial peptides: pore formers or metabolic inhibitors in bacteria?. Nat Rev Microbiol.

[136] Jenssen H, Hamill P, Hancock RE (2006). Peptide antimicrobial agents. Clin Microbiol Rev.

[137] Hancock RE, Rozek A (2002). Role of membranes in the activities of antimicrobial cationic peptides. FEMS Microbiol Lett.

[138] Sandgren S, Wittrup A, Cheng F, Jonsson M, Eklund E, Busch S, Belting M (2004). The human antimicrobial peptide LL-37 transfers extracellular DNA plasmid to the nuclear compartment of mammalian cells via lipid rafts and proteoglycan-dependent endocytosis. J Biol Chem.

[139] Zhang X, Oglecka K, Sandgren S, Belting M, Esbjorner EK, Norden B, Graslund A (2010). Dual functions of the human antimicrobial peptide LL-37-target membrane perturbation and host cell cargo delivery. Biochim Biophys Acta.

[140] Moir RD, Lathe R, Tanzi RE (2018). The antimicrobial protection hypothesis of Alzheimer’s disease. Alzheimer’s Dement.

[141] Mathura VS, Paris D, Ait-Ghezala G, Quadros A, Patel NS, Kolippakkam DN, Volmar CH, Mullan MJ (2005). Model of Alzheimer’s disease amyloid-beta peptide based on a RNA binding protein. Biochem Biophys Res Commun.

[142] Hegde ML, Anitha S, Latha KS, Mustak MS, Stein R, Ravid R, Rao KS (2004). First evidence for helical transitions in supercoiled DNA by amyloid Beta Peptide (1-42) and aluminum: a new insight in understanding Alzheimer’s disease. J Mol Neurosci.

[143] Yu H, Ren J, Qu X (2007). Time-dependent DNA condensation induced by amyloid beta-peptide. Biophys J.

[144] Barrantes A, Rejas MT, Benitez MJ, Jimenez JS (2007). Interaction between Alzheimer’s Abeta1-42 peptide and DNA detected by surface plasmon resonance. J Alzheimers Dis.

[145] Geng J, Zhao C, Ren J, Qu X (2010). Alzheimer’s disease amyloid beta converting left-handed Z-DNA back to right-handed B-form. Chem Commun (Camb).

[146] Camero S, Ayuso JM, Barrantes A, Benitez MJ, Jimenez JS (2013). Specific binding of DNA to aggregated forms of Alzheimer’s disease amyloid peptides. Int J Biol Macromol.

[147] Maloney B, Lahiri DK (2011). The Alzheimer’s amyloid beta-peptide (Abeta) binds a specific DNA Abeta-interacting domain (AbetaID) in the APP, BACE1, and APOE promoters in a sequence-specific manner: characterizing a new regulatory motif. Gene.

[148] Mangé A, Crozet C, Lehmann S, Beranger F (2004). Scrapie-like prion protein is translocated to the nuclei of infected cells independently of proteasome inhibition and interacts with chromatin. J Cell Sci.

[149] Lathe R, Darlix JL (2017). Prion protein PRNP: a new player in innate immunity? - The Abeta connection. J Alzheimers Dis Rep.

[150] Hegde RS, Mastrianni JA, Scott MR, DeFea KA, Tremblay P, Torchia M, DeArmond SJ, Prusiner SB, Lingappa VR (1998). A transmembrane form of the prion protein in neurodegenerative disease. Science.

[151] Baron GS, Wehrly K, Dorward DW, Chesebro B, Caughey B (2002). Conversion of raft associated prion protein to the protease-resistant state requires insertion of PrP-res (PrP(Sc)) into contiguous membranes. EMBO J.

[152] Sanghera N, Pinheiro TJ (2002). Binding of prion protein to lipid membranes and implications for prion conversion. J Mol Biol.

[153] Critchley P, Kazlauskaite J, Eason R, Pinheiro TJ (2004). Binding of prion proteins to lipid membranes. Biochem Biophys Res Commun.

[154] Zhong J, Zheng W, Huang L, Hong Y, Wang L, Qiu Y, Sha Y (2007). PrP106-126 amide causes the semi-penetrated poration in the supported lipid bilayers. Biochim Biophys Acta.

[155] Shin JI, Shin JY, Kim JS, Yang YS, Shin YK, Kweon DH (2008). Deep membrane insertion of prion protein upon reduction of disulfide bond. Biochem Biophys Res Commun.

[156] Piersanti S, Martina Y, Cherubini G, Avitabile D, Saggio I (2004). Use of DNA microarrays to monitor host response to virus and virus-derived gene therapy vectors. Am J Pharmacogenomics.

[157] Caruso P, Burla R, Piersanti S, Cherubini G, Remoli C, Martina Y, Saggio I (2009). Prion expression is activated by adenovirus 5 infection and affects the adenoviral cycle in human cells. Virology.

[158] Yuan J, Cahir-McFarland E, Zhao B, Kieff E (2006). Virus and cell RNAs expressed during Epstein–Barr virus replication. J Virol.

[159] Walters KA, Joyce MA, Thompson JC, Smith MW, Yeh MM, Proll S, Zhu LF, Gao TJ, Kneteman NM, Tyrrell DL, Katze MG (2006). Host-specific response to HCV infection in the chimeric SCID-beige/Alb-uPA mouse model: role of the innate antiviral immune response. PLoS Pathog.

[160] Hojka-Osinska A, Budzko L, Zmienko A, Rybarczyk A, Maillard P, Budkowska A, Figlerowicz M, Jackowiak P (2016). RNA-Seq-based analysis of differential gene expression associated with hepatitis C virus infection in a cell culture. Acta Biochim Pol.

[161] Muller WE, Pfeifer K, Forrest J, Rytik PG, Eremin VF, Popov SA, Schroder HC (1992). Accumulation of transcripts coding for prion protein in human astrocytes during infection with human immunodeficiency virus. Biochim Biophys Acta.

[162] Konturek PC, Bazela K, Kukharskyy V, Bauer M, Hahn EG, Schuppan D (2005). Helicobacter pylori upregulates prion protein expression in gastric mucosa: a possible link to prion disease. World J Gastroenterol.

[163] Ding T, Zhou X, Kouadir M, Shi F, Yang Y, Liu J, Wang M, Yin X, Yang L, Zhao D (2013). Cellular prion protein participates in the regulation of inflammatory response and apoptosis in BV2 microglia during infection with Mycobacterium bovis. J Mol Neurosci.

[164] Lotscher M, Recher M, Hunziker L, Klein MA (2003). Immunologically induced, complement-dependent up-regulation of the prion protein in the mouse spleen: follicular dendritic cells versus capsule and trabeculae. J Immunol.

[165] Roberts TK, Eugenin EA, Morgello S, Clements JE, Zink MC, Berman JW (2010). PrPC, the cellular isoform of the human prion protein, is a novel biomarker of HIV-associated neurocognitive impairment and mediates neuroinflammation. Am J Pathol.

[166] Stanton JB, Knowles DP, O’Rourke KI, Herrmann-Hoesing LM, Mathison BA, Baszler TV (2008). Small-ruminant lentivirus enhances PrPSc accumulation in cultured sheep microglial cells. J Virol.

[167] Pasupuleti M, Roupe M, Rydengard V, Surewicz K, Surewicz WK, Chalupka A, Malmsten M, Sorensen OE, Schmidtchen A (2009). Antimicrobial activity of human prion protein is mediated by its N-terminal region. PLoS One.

[168] Linden R, Martins VR, Prado MA, Cammarota M, Izquierdo I, Brentani RR (2008). Physiology of the prion protein. Physiol Rev.

[169] Kumar Deepak Kumar Vijaya, Choi Se Hoon, Washicosky Kevin J., Eimer William A., Tucker Stephanie, Ghofrani Jessica, Lefkowitz Aaron, McColl Gawain, Goldstein Lee E., Tanzi Rudolph E., Moir Robert D. (2016). Amyloid-β peptide protects against microbial infection in mouse and worm models of Alzheimer’s disease. Science Translational Medicine.

[170] Leblanc P, Baas D, Darlix JL (2004). Analysis of the interactions between HIV-1 and the cellular prion protein in a human cell line. J Mol Biol.

[171] Alais S, Soto-Rifo R, Balter V, Gruffat H, Manet E, Schaeffer L, Darlix JL, Cimarelli A, Raposo G, Ohlmann T, Leblanc P (2012). Functional mechanisms of the cellular prion protein (PrP(C)) associated anti-HIV-1 properties. Cell Mol Life Sci.

[172] Kagan BL (2011). Antimicrobial amyloids?. Biophys J.

[173] Kagan BL, Jang H, Capone R, Teran AF, Ramachandran S, Lal R, Nussinov R (2012). Antimicrobial properties of amyloid peptides. Mol Pharm.

[174] Deleault NR, Lucassen RW, Supattapone S (2003). RNA molecules stimulate prion protein conversion. Nature.

[175] Supattapone S (2014). Synthesis of high titer infectious prions with cofactor molecules. J Biol Chem.

[176] Deleault NR, Kascsak R, Geoghegan JC, Supattapone S (2010). Species-dependent differences in cofactor utilization for formation of the protease-resistant prion protein in vitro. Biochemistry.

[177] Zeiler B, Adler V, Kryukov V, Grossman A (2003). Concentration and removal of prion proteins from biological solutions. Biotechnol Appl Biochem.

[178] Adler V, Zeiler B, Kryukov V, Kascsak R, Rubenstein R, Grossman A (2003). Small, highly structured RNAs participate in the conversion of human recombinant PrP(Sen) to PrP(Res) in vitro. J Mol Biol.

[179] Macedo B, Cordeiro Y (2017) Unraveling prion protein interactions with aptamers and other PrP-binding nucleic acids. Int J Mol Sci 18 10.3390/ijms18051023 PMC5454936 28513534

[180] Bera A, Biring S (2018). A quantitative characterization of interaction between prion protein with nucleic acids. Biochem Biophys Rep.

[181] Ogawa Y, Kawamura T, Matsuzawa T, Aoki R, Gee P, Yamashita A, Moriishi K, Yamasaki K, Koyanagi Y, Blauvelt A, Shimada S (2013). Antimicrobial peptide LL-37 produced by HSV-2-infected keratinocytes enhances HIV infection of Langerhans cells. Cell Host Microbe.

[182] Wojtowicz WM, Farzan M, Joyal JL, Carter K, Babcock GJ, Israel DI, Sodroski J, Mirzabekov T (2002). Stimulation of enveloped virus infection by beta-amyloid fibrils. J Biol Chem.

[183] Cheng SB, Ferland P, Webster P, Bearer EL (2011). Herpes simplex virus dances with amyloid precursor protein while exiting the cell. PLoS One.

[184] Castellano LM, Shorter J (2012). The surprising role of amyloid fibrils in HIV infection. Biology (Basel).

[185] Tang Q, Roan NR, Yamamura Y (2013). Seminal plasma and semen amyloids enhance cytomegalovirus infection in cell culture. J Virol.

[186] Zhang H, Gao S, Pei R, Chen X, Li C (2017). Hepatitis C virus-induced prion protein expression facilitates hepatitis C virus replication. Virol Sin.

[187] Kim BH, Shin HY, Goto JJ, Carp RI, Choi EK, Kim YS (2016). Cellular prion protein combined with Galectin-3 and -6 affects the infectivity titer of an endogenous retrovirus assayed in hippocampal neuronal cells. PLoS One.

[188] Lee KH, Jeong BH, Jin JK, Meeker HC, Kim JI, Carp RI, Kim YS (2006). Scrapie infection activates the replication of ecotropic, xenotropic, and polytropic murine leukemia virus (MuLV) in brains and spinal cords of senescence-accelerated mice: implication of MuLV in progression of scrapie pathogenesis. Biochem Biophys Res Commun.

[189] Leblanc P, Alais S, Porto-Carreiro I, Lehmann S, Grassi J, Raposo G, Darlix JL (2006). Retrovirus infection strongly enhances scrapie infectivity release in cell culture. EMBO J.

[190] Carp RI, Meeker HC, Caruso V, Sersen E (1999). Scrapie strain-specific interactions with endogenous murine leukaemia virus. J Gen Virol.

[191] Han JS, Boeke JD (2005). LINE-1 retrotransposons: modulators of quantity and quality of mammalian gene expression?. BioEssays.

[192] Muotri AR, Chu VT, Marchetto MC, Deng W, Moran JV, Gage FH (2005). Somatic mosaicism in neuronal precursor cells mediated by L1 retrotransposition. Nature.

[193] Coufal NG, Garcia-Perez JL, Peng GE, Yeo GW, Mu Y, Lovci MT, Morell M, O’Shea KS, Moran JV, Gage FH (2009). L1 retrotransposition in human neural progenitor cells. Nature.

[194] Baillie JK, Barnett MW, Upton KR, Gerhardt DJ, Richmond TA, De SF, Brennan PM, Rizzu P, Smith S, Fell M, Talbot RT, Gustincich S, Freeman TC, Mattick JS, Hume DA, Heutink P, Carninci P, Jeddeloh JA, Faulkner GJ (2011). Somatic retrotransposition alters the genetic landscape of the human brain. Nature.

[195] Akagi K, Li J, Stephens RM, Volfovsky N, Symer DE (2008). Extensive variation between inbred mouse strains due to endogenous L1 retrotransposition. Genome Res.

[196] Kambere MB, Lane RP (2009). Exceptional LINE density at V1R loci: the Lyon repeat hypothesis revisited on autosomes. J Mol Evol.

[197] Ostertag EM, Kazazian HH (2001). Biology of mammalian L1 retrotransposons. Annu Rev Genet.

[198] Waters PD, Dobigny G, Waddell PJ, Robinson TJ (2007). Evolutionary history of LINE-1 in the major clades of placental mammals. PLoS One.

[199] Sutkowski N, Conrad B, Thorley-Lawson DA, Huber BT (2001). Epstein-Barr virus transactivates the human endogenous retrovirus HERV-K18 that encodes a superantigen. Immunity.

[200] Kwun HJ, Han HJ, Lee WJ, Kim HS, Jang KL (2002). Transactivation of the human endogenous retrovirus K long terminal repeat by herpes simplex virus type 1 immediate early protein 0. Virus Res.

[201] Ruprecht K, Obojes K, Wengel V, Gronen F, Kim KS, Perron H, Schneider-Schaulies J, Rieckmann P (2006). Regulation of human endogenous retrovirus W protein expression by herpes simplex virus type 1: implications for multiple sclerosis. J Neurovirol.

[202] Brudek T, Luhdorf P, Christensen T, Hansen HJ, Moller-Larsen A (2007). Activation of endogenous retrovirus reverse transcriptase in multiple sclerosis patient lymphocytes by inactivated HSV-1, HHV-6 and VZV. J Neuroimmunol.

[203] Tai AK, Luka J, Ablashi D, Huber BT (2009). HHV-6A infection induces expression of HERV-K18-encoded superantigen. J Clin Virol.

[204] Hsiao FC, Tai AK, Deglon A, Sutkowski N, Longnecker R, Huber BT (2009). EBV LMP-2A employs a novel mechanism to transactivate the HERV-K18 superantigen through its ITAM. Virology.

[205] Mameli G, Poddighe L, Mei A, Uleri E, Sotgiu S, Serra C, Manetti R, Dolei A (2012). Expression and activation by Epstein Barr virus of human endogenous retroviruses-W in blood cells and astrocytes: inference for multiple sclerosis. PLoS One.

[206] Bergallo M, Galliano I, Montanari P, Gambarino S, Mareschi K, Ferro F, Fagioli F, Tovo PA, Ravanini P (2015). CMV induces HERV-K and HERV-W expression in kidney transplant recipients. J Clin Virol.

[207] Jang KL, Latchman DS (1989). HSV infection induces increased transcription of Alu repeated sequences by RNA polymerase III. FEBS Lett.

[208] Panning B, Smiley JR (1989). Regulation of cellular genes transduced by herpes simplex virus. J Virol.

[209] Jang KL, Latchman DS (1992). The herpes simplex virus immediate-early protein ICP27 stimulates the transcription of cellular Alu repeated sequences by increasing the activity of transcription factor TFIIIC. Biochem J.

[210] Karijolich J, Abernathy E, Glaunsinger BA (2015). Infection-induced retrotransposon-derived noncoding RNAs enhance herpesviral gene expression via the NF-kappaB pathway. PLoS Pathog.

[211] Kocisko DA, Vaillant A, Lee KS, Arnold KM, Bertholet N, Race RE, Olsen EA, Juteau JM, Caughey B (2006). Potent antiscrapie activities of degenerate phosphorothioate oligonucleotides. Antimicrob Agents Chemother.

[212] Magzoub M, Sandgren S, Lundberg P, Oglecka K, Lilja J, Wittrup A, Goran Eriksson LE, Langel U, Belting M, Graslund A (2006). N-terminal peptides from unprocessed prion proteins enter cells by macropinocytosis. Biochem Biophys Res Commun.

[213] Yin S, Fan X, Yu S, Li C, Sy MS (2008). Binding of recombinant but not endogenous prion protein to DNA causes DNA internalization and expression in mammalian cells. J Biol Chem.

[214] Rieger R, Edenhofer F, Lasmezas CI, Weiss S (1997). The human 37-kDa laminin receptor precursor interacts with the prion protein in eukaryotic cells. Nat Med.

[215] Morel E, Andrieu T, Casagrande F, Gauczynski S, Weiss S, Grassi J, Rousset M, Dormont D, Chambaz J (2005). Bovine prion is endocytosed by human enterocytes via the 37 kDa/67 kDa laminin receptor. Am J Pathol.

[216] Da Costa Dias B, Jovanovic K, Gonsalves D, Moodley K, Reusch U, Knackmuss S, Weinberg MS, Little M, Weiss SF (2014). The 37 kDa/67 kDa laminin receptor acts as a receptor for Abeta42 internalization. Sci Rep.

[217] Fevrier B, Vilette D, Archer F, Loew D, Faigle W, Vidal M, Laude H, Raposo G (2004). Cells release prions in association with exosomes. Proc Natl Acad Sci U S A.

[218] Kim KM, Abdelmohsen K, Mustapic M, Kapogiannis D, Gorospe M (2017). RNA in extracellular vesicles. Wiley Interdiscip Rev RNA.

[219] Mateescu B, Kowal EJ, van Balkom BW, Bartel S, Bhattacharyya SN, Buzas EI, Buck AH, Chow FW, Das S, Driedonks TA, Fernandez-Messina L, Haderk F, Hill AF, Jones JC, Van Keuren-Jensen KR, Lai CP, Lasser C, Liegro ID, Lunavat TR, Lorenowicz MJ, Maas SL, Mager I, Mittelbrunn M, Momma S, Mukherjee K, Nawaz M, Pegtel DM, Pfaffl MW, Schiffelers RM, Tahara H, Thery C, Tosar JP, Wauben MH, Witwer KW, Nolte-’t Hoen EN (2017). Obstacles and opportunities in the functional analysis of extracellular vesicle. J Extracell Vesicles.

[220] Vella LJ, Sharples RA, Lawson VA, Masters CL, Cappai R, Hill AF (2007). Packaging of prions into exosomes is associated with a novel pathway of PrP processing. J Pathol.

[221] Bellingham SA, Coleman BM, Hill AF (2012). Small RNA deep sequencing reveals a distinct miRNA signature released in exosomes from prion-infected neuronal cells. Nucleic Acids Res.

[222] Booth AM, Fang Y, Fallon JK, Yang JM, Hildreth JE, Gould SJ (2006). Exosomes and HIV Gag bud from endosome-like domains of the T cell plasma membrane. J Cell Biol.

[223] Maldonado JO, Martin JL, Mueller JD, Zhang W, Mansky LM (2014). New insights into retroviral Gag–Gag and Gag-membrane interactions. Front Microbiol.

[224] Coffin JM, Fan H (2016). The discovery of reverse transcriptase. Annu Rev Virol.

[225] Kato M, Han TW, Xie S, Shi K, Du X, Wu LC, Mirzaei H, Goldsmith EJ, Longgood J, Pei J, Grishin NV, Frantz DE, Schneider JW, Chen S, Li L, Sawaya MR, Eisenberg D, Tycko R, McKnight SL (2012). Cell-free formation of RNA granules: low complexity sequence domains form dynamic fibers within hydrogels. Cell.

[226] Yuan J, Xiao X, McGeehan J, Dong Z, Cali I, Fujioka H, Kong Q, Kneale G, Gambetti P, Zou WQ (2006). Insoluble aggregates and protease-resistant conformers of prion protein in uninfected human brains. J Biol Chem.

[227] Scheinker VS, Lozovskaya ER, Bishop JG, Corces VG, Evgen’ev MB (1990). A long terminal repeat-containing retrotransposon is mobilized during hybrid dysgenesis in Drosophila virilis. Proc Natl Acad Sci USA.

[228] Vieira J, Vieira CP, Hartl DL, Lozovskaya ER (1998). Factors contributing to the hybrid dysgenesis syndrome in *Drosophila virilis*. Genet Res.

[229] Kordis D, Gubensek F (1998). Unusual horizontal transfer of a long interspersed nuclear element between distant vertebrate classes. Proc Natl Acad Sci USA.

[230] Kordis D, Gubensek F (1999). Horizontal transfer of non-LTR retrotransposons in vertebrates. Genetica.

[231] Ivancevic AM, Kortschak RD, Bertozzi T, Adelson DL (2018). Horizontal transfer of BovB and L1 retrotransposons in eukaryotes. Genome Biol.

[232] Bodea GO, McKelvey EGZ, Faulkner GJ (2018). Retrotransposon-induced mosaicism in the neural genome. Open Biol.

[233] Suarez NA, Macia A, Muotri AR (2018). LINE-1 retrotransposons in healthy and diseased human brain. Dev Neurobiol.

[234] Sun W, Samimi H, Gamez M, Zare H, Frost B (2018). Pathogenic tau-induced piRNA depletion promotes neuronal death through transposable element dysregulation in neurodegenerative tauopathies. Nat Neurosci.

[235] Silva Jerson L., Lima Luis Mauricio T.R., Foguel Debora, Cordeiro Yraima (2009). Response to Radulescu and Brenig: Infectious nucleic acids in prion disease: halfway there. Trends in Biochemical Sciences.

[236] Zahn R, Liu A, Luhrs T, Riek R, Von SC, Lopez GF, Billeter M, Calzolai L, Wider G, Wuthrich K (2000). NMR solution structure of the human prion protein. Proc Natl Acad Sci USA.

[237] Chen SG, Zou W, Parchi P, Gambetti P (2000) PrPSc typing by N-terminal sequencing and mass spectrometry. Arch Virol Suppl 209–216 11214924

[238] Pan T, Li R, Kang SC, Wong BS, Wisniewski T, Sy MS (2004). Epitope scanning reveals gain and loss of strain specific antibody binding epitopes associated with the conversion of normal cellular prion to scrapie prion. J Neurochem.

[239] Thuring CM, Erkens JH, Jacobs JG, Bossers A, Van Keulen LJ, Garssen GJ, Van Zijderveld FG, Ryder SJ, Groschup MH, Sweeney T, Langeveld JP (2004). Discrimination between scrapie and bovine spongiform encephalopathy in sheep by molecular size, immunoreactivity, and glycoprofile of prion protein. J Clin Microbiol.

[240] Saijo E, Hughson AG, Raymond GJ, Suzuki A, Horiuchi M, Caughey B (2016). PrPSc-specific antibody reveals C-terminal conformational differences between prion strains. J Virol.

[241] Lund C, Olsen CM, Tveit H, Tranulis MA (2007). Characterization of the prion protein 3F4 epitope and its use as a molecular tag. J Neurosci Methods.

[242] Lathe R, Harris A (2009). Differential display detects host nucleic acid motifs altered in scrapie-infected brain. J Mol Biol.

[243] Skinner PJ, Abbassi H, Chesebro B, Race RE, Reilly C, Haase AT (2006). Gene expression alterations in brains of mice infected with three strains of scrapie. BMC Genomics.

[244] Gordon PM, Schutz E, Beck J, Urnovitz HB, Graham C, Clark R, Dudas S, Czub S, Sensen M, Brenig B, Groschup MH, Church RB, Sensen CW (2009). Disease-specific motifs can be identified in circulating nucleic acids from live elk and cattle infected with transmissible spongiform encephalopathies. Nucleic Acids Res.

[245] Greenwood AD, Vincendeau M, Schmadicke AC, Montag J, Seifarth W, Motzkus D (2011). Bovine spongiform encephalopathy infection alters endogenous retrovirus expression in distinct brain regions of cynomolgus macaques (*Macaca fascicularis*). Mol Neurodegener.

[246] Murdoch GH, Sklaviadis T, Manuelidis EE, Manuelidis L (1990). Potential retroviral RNAs in Creutzfeldt-Jakob disease. J Virol.

[247] Akowitz A, Manuelidis EE, Manuelidis L (1993). Protected endogenous retroviral sequences copurify with infectivity in experimental Creutzfeldt-Jakob disease. Arch Virol.

[248] Jeong BH, Lee YJ, Carp RI, Kim YS (2010). The prevalence of human endogenous retroviruses in cerebrospinal fluids from patients with sporadic Creutzfeldt–Jakob disease. J Clin Virol.

[249] Schutz E, Urnovitz HB, Iakoubov L, Schulz-Schaeffer W, Wemheuer W, Brenig B (2005). Bov-tA short interspersed nucleotide element sequences in circulating nucleic acids from sera of cattle with bovine spongiform encephalopathy (BSE) and sera of cattle exposed to BSE. Clin Diagn Lab Immunol.

[250] Stengel A, Bach C, Vorberg I, Frank O, Gilch S, Lutzny G, Seifarth W, Erfle V, Maas E, Schatzl H, Leib-Mosch C, Greenwood AD (2006). Prion infection influences murine endogenous retrovirus expression in neuronal cells. Biochem Biophys Res Commun.

[251] Barnard E, Estibeiro K, Duncan R, Baird J, Fettes D, Wood J, Fraser H, Estibeiro P, Lathe R (2019) Possible origin of the scrapie genome in small endogenous RNAs; studies on eight candidate species in 263 K scrapie-infected hamster brain. BioRxiv. Published online December 5, 2019

